# Characteristics of interprofessional collaboration between physicians and chiropractors within Québec multidisciplinary clinics: a mixed-methods study

**DOI:** 10.1186/s12998-026-00673-9

**Published:** 2026-09-05

**Authors:** Marc-André Blanchette, Lilly Nguyen, Jean-Philip Hudon-Dionne, Geneviève Lavigne, Marie-Andrée Mercier, Jason W. Busse

**Affiliations:** 1 Département chiropratique, Université du Québec à Trois-Rivières, 3351boul. des Forges, C.P. 500, Trois-Rivières, QC G9A 5H7 Canada; 2 Département des sciences de la santé, Université du Québec en Abitibi-Témiscamingue (UQAT), Rouyn-Noranda, QC Canada; 3 Centre de recherche du CHU de Québec – Université Laval, Axe Santé des populations et pratiques optimales en santé, Quebec, Quebec Canada; 4 Advisor, Ordre des chiropraticiens du Québec, Montréal, Québec, Canada; 5 Private practice, Vertebralis chiropratique, Rivière-du-Loup Quebec, Canada; 6 Department of Anesthesia, McMaster University, Hamilton ON, Canada

**Keywords:** Interprofessional relations, Chiropractic, Physicians, Referral and consultation, Delivery of health care, Integrated, Primary health care

## Abstract

**Background:**

Physician referral rates to chiropractors remain low in Quebec, and few multidisciplinary clinics include both professions. Yet these settings represent a unique opportunity to study interprofessional collaboration in musculoskeletal care. This study aimed to identify facilitators and barriers to physician-chiropractor collaboration in multidisciplinary clinic settings, and to evaluate perceptions regarding the effectiveness of collaborative efforts on patient care.

**Methods:**

An explanatory sequential mixed-methods design was employed. Between February and November 2024, quantitative data were collected via surveys of 23 chiropractors and 19 physicians practising in multidisciplinary clinics including both professions. Qualitative data were collected through a semi-structured focus group with seven chiropractors and individual semi-structured interviews with five physicians. Survey data were reported with descriptive statistics, and qualitative data were analyzed using thematic analysis.

**Results:**

Survey results indicated that nearly 70% of physicians referred patients to chiropractors at least monthly, driven primarily by established relationships or patient requests. Despite broadly positive mutual attitudes, physicians expressed concerns about the safety of cervical manipulation and professional liability. Chiropractors identified personal knowledge of other providers and physical proximity as the strongest enablers of collaboration with physicians, while clinical workload and patients’ financial constraints were the primary barriers. Both professional groups attributed high value to interprofessional collaboration; most chiropractors and physicians reported that collaboration improved patient satisfaction, clinical outcomes, and overall quality of care. Qualitative analysis revealed three themes: perception, organization of care and services, and communication. Perceptual barriers included educational silos, historical professional tensions, and variability in chiropractic practices. Structural obstacles included out-of-pocket costs, exclusion from public care settings, and administrative burdens. Facilitators included personal experience with chiropractic care, co-location, clear referral pathways, and direct communication modalities.

**Conclusions:**

In multidisciplinary clinic settings, interprofessional collaboration between physicians and chiropractors is valued and perceived as beneficial by both professional groups. Despite a favourable relational environment, collaborative practice remains heavily reliant on informal, relationship-driven interactions rather than embedded organizational structures. Our findings underscore the need for education, organizational integration, and communication infrastructure to improve interprofessional musculoskeletal care.

**Supplementary Information:**

The online version contains supplementary material available at https://doi.org/10.1186/s12998-026-00673-9.

## Introduction

Musculoskeletal disorders, particularly low back pain, represent a leading cause of disability worldwide, affecting over 600 million individuals in 2020 and accounting for the greatest number of years lived with disability [1]. The lifetime prevalence of back pain among Canadians is approximately 84% [2], a burden that contributes substantially to healthcare costs and lost productivity [3–5]. Effective management of musculoskeletal conditions is a clinical and an economic imperative, driving the need for care models that optimize timely access, clinical outcomes, and resource utilization.

In response to this burden, interprofessional collaboration in primary care, defined as “the process in which different professional groups work together to positively impact healthcare” [6], has emerged as a promising strategy. Within this collaborative approach, chiropractors are recognized as key partners in the management of musculoskeletal disorders [7, 8]. Several case studies and pilot programmes have suggested that embedding chiropractors into primary-care teams can improve patient outcomes, reduce reliance on opioids, and improve physicians’ perceptions of chiropractic care [7–18]. However, the incorporation of collaborative care models between physicians and chiropractors into routine practice remains limited.

Surveys of Canadian physicians have found that 82 to 86% believe chiropractors provide effective care for musculoskeletal conditions, but the majority also have concerns regarding safety of cervical manipulation and variability in practice approaches [19–22]. Previous studies similarly highlight educational silos and historical tensions as persistent barriers to interprofessional collaboration [23, 24]. Furthermore, chiropractors in Canada predominantly operate in private, fee-for‐service practices [25, 26], a funding model that may hinder collaborative engagement compared with capitation or blended remuneration schemes [7, 8, 27].

In Quebec, chiropractors receive an average of 10 patients referred by a physician per year while chiropractors from other provinces receive an annual average of 14 (Manitoba) to 28 (Atlantic provinces) patients [25]. This provincial variation underscores the need to examine local practice ecosystems, where funding mechanisms, regulatory frameworks, and interprofessional cultures may diverge from national norms. Emerging evidence suggests that collaborative models involving physicians and chiropractors could reduce opioid prescriptions, unnecessary imaging, and inappropriate surgical consultations for musculoskeletal disorders [16, 17, 28, 29]. Such findings align with broader efforts to curb low-value care and promote non‐pharmacological interventions as first‐line treatment for spine pain [30].

Evidence on practices, perceptions, and organizational factors shaping chiropractor–physician collaboration in Quebec remains sparse. Most existing studies focus on pilot initiatives or single-site evaluations, limiting generalizability and leaving critical questions unanswered. For instance, we lack robust information on: (1) the frequency and modalities of interprofessional interactions in multidisciplinary environments; (2) the attitudinal alignment between chiropractors and physicians regarding collaborative roles, responsibilities, and outcomes; and (3) the structural enablers and impediments within multidisciplinary care settings. A detailed understanding of how chiropractors and physicians currently collaborate in multidisciplinary environments is important to inform targeted interventions to embed interprofessional care more systematically. Moreover, mapping perceived barriers and facilitators across multiple perceptual, organizational, and communicative dimensions will help interest holders prioritize strategies that yield the greatest impact on patient outcomes, provider satisfaction, and health‐system efficiency.

The present study employs an explanatory sequential mixed-methods design to explore interprofessional collaboration practices between chiropractors and physicians working in a multidisciplinary environment within Quebec, Canada. By integrating quantitative survey data from practising clinicians with qualitative insights from focus groups and semi‐structured interviews, we sought to identify facilitating factors and barriers to collaboration between chiropractors and physicians in multidisciplinary clinic settings, and to evaluate perceptions regarding the effectiveness of collaborative efforts on patient care.

We draw on the conceptual framework of Mior and colleagues [1] for collaborative musculoskeletal care between chiropractors and physicians in primary care. This framework identifies three process-based categories necessary to optimize collaboration: communication (formal and informal exchanges, shared records, patient information tools), practice parameters (scope of practice, evidence-based care, guidelines), and service delivery (access, affordability, reimbursement, liability). Because our themes were derived inductively, we used this framework not to structure the analysis but as a lens for interpreting and comparing the determinants of collaboration we identified.

## Methods

This mixed-methods study employed quantitative and qualitative approaches to explore collaborative practices between physicians and chiropractors working in a common multidisciplinary practice in Quebec, Canada. Ethics approval was obtained from the Research Ethics Board of the Université du Québec à Trois-Rivières (CER-23-303-07.04).

### Study design

Our study comprised four components: (1) a survey of chiropractors working with physicians in a multidisciplinary environment; (2) a survey of physicians working with chiropractors in a multidisciplinary environment; (3) a focus group semi-structured discussion with chiropractors; and (4) individual semi-structured interviews with physicians. This mixed-methods design enabled integration across professional groups and methodological approaches, thereby enhancing the credibility and depth of the findings. The design was explanatory sequential: the quantitative surveys were conducted and analysed first, and the qualitative strand (the chiropractor focus group and the physician interviews) was then used to explain and enrich survey findings.

### Study population and recruitment

#### Chiropractor survey

The target population for the first component of our study consisted of chiropractors practising in multidisciplinary clinics. Eligible chiropractors were licensed in the province of Québec and practising in a clinic that included at least one physician. An invitation was sent to the more than 1350 members of the *Ordre des chiropraticiens du Québec* (provincial chiropractic regulatory board) through their July 2023 newsletter. The registry of the provincial chiropractic regulatory board was also screened to identify multidisciplinary clinics. We employed snowball sampling to recruit additional eligible chiropractors. Each potential participant received a personalized email invitation followed by up to three weekly reminders. The online survey was opened from February 21, 2024, to April 26, 2024.

#### Physician survey

The target population for the second component consisted of physicians practising in multidisciplinary clinics involving at least one chiropractor. Eligible physicians were licensed in the province of Québec and practising in a clinic that included at least one chiropractor. We identified 137 physicians by consulting websites of multidisciplinary clinics where eligible chiropractors had a practicing address. Between 21 and 29 February 2024, each identified physician received an invitation to participate in our study along with a paper version of our questionnaire by mail, since their email addresses were not publicly available.

#### Chiropractor Focus Group

All chiropractors invited to complete our survey were also invited to participate in a one-hour virtual focus group on April 18, 2024. Participants were credited with one hour of continuing education for their participation. We aimed to recruit between seven and ten participants to foster dynamic discussion, stimulate ideas that might not emerge in isolation, and generate richer reflections on interprofessional collaboration [31, 32].

#### Physician interviews

Physicians were recruited for interviews through two channels: physicians who indicated willingness at the end of the survey were invited directly, and eligible chiropractors were asked to forward the interview invitation to physicians with whom they collaborated. Interviews were one-hour individual semi-structured sessions (between May 4th and November 14, 2024). We aimed to conduct between 5 and 10 interviews to capture diverse physician perspectives on interprofessional collaboration. We provided individual interviews with flexible scheduling to reduce barriers to participation.

### Data collection

#### Surveys

Survey data were collected via an online survey tool developed by the Université du Québec à Trois-Rivières (https://confluence.uqtr.ca/display/AOPSP/BIQ). For the chiropractor survey, we collected all data directly via the online platform. Physician survey responses, distributed by mail, were subsequently entered into the same platform by members of our research team. The chiropractor survey was composed of the questionnaire developed by Perreault and colleagues [33, 34], adapted to the context of Quebec’s chiropractic practice. The physician survey was composed of questionnaires developed by Busse et al. [22], and Gillespie et al. [35], each similarly adapted. For example, physiotherapy and pharmacy were replaced by chiropractic, and questions that did not align with our research objective were omitted to produce parsimonious questionnaires (Supplementary file 1).

#### Focus group with chiropractors

The 60-minute focus group with chiropractors was conducted with Microsoft Teams. Semi-structured questions, adapted from Perreault’s work [36], guided the exploration of collaboration between chiropractors and physicians as well as identification of key organizational factors influencing collaboration. The session was recorded and corrected against the video recording by one of the authors. Field notes were taken by the facilitator during and immediately following the focus group to capture observations and initial impressions. Transcripts were not returned to participants for review.

#### Individual semi-structured interviews

Individual semi-structured 60-minutes interviews with physicians were conducted via the Microsoft Teams platform. The interview guide was adapted from Perreault’s work [36] in light of the findings from the surveys and the focus group with chiropractors. Interviews were also recorded and transcribed verbatim. As for the focus group, field notes were taken by the interviewer, and participants did not review transcripts.

### Data analysis

#### Quantitative analysis

Descriptive statistics (means and standard deviations for normally distributed continuous data; median and interquartile rage [IQR] for non-normally distributed continuous data; and frequencies and percentages for categorical data) were computed for all survey questions using IBM SPSS version 29.

#### Qualitative analysis

Focus group and interview transcripts were analyzed using thematic analysis [37]. Two members of the research team independently coded all transcripts, generating initial codes grounded in the data. Codes were iteratively reviewed and grouped into candidate themes and subthemes through a process of constant comparison across participants and data sources. Discrepancies between coders were resolved through discussion until consensus was reached. Researcher reflexivity was evaluated for potential impact on the interpretation of results (see Supplementary file 2). Qualitative data were managed and analyzed manually, without the use of qualitative data analysis software. This study was reported in accordance with the Consolidated Criteria for Reporting Qualitative Research (COREQ) [38].

#### Mixed-methods integration and reporting

Coding was inductive: initial codes were generated directly from the data (de novo) rather than derived from an a priori framework. Consistent with the explanatory sequential design, the quantitative and qualitative strands were analysed separately and then integrated at the interpretation stage using a joint display. The display is organized by meta-inference: for each integrated dimension, the corresponding quantitative result and qualitative finding were placed side by side, and a meta-inference characterizing the fit between strands as confirmation, expansion, or discordance was generated [39–41].

## Results

### Survey results

#### Demographic and practice characteristics

Of the 33 chiropractors and 137 physicians invited, 23 chiropractors (69.7%) and 19 physicians (13.9%) completed our surveys. Among chiropractors, 52% were female with an average of 12.8 years in practice (SD = 10.1 years), working an average of 29.8 h weekly (SD = 12.1 h/week), and treating an average of 58 patients per week (SD = 31.9 patients/week). Most chiropractors operated as independent contractors (65%) most often remunerated under a fee-for-service model (70%) and practised in a building with other health-related business or organizations (44%). The remaining chiropractors reported an hourly salary (13%), a mixed salary and fee-for-service model (4%), or another arrangement (4%); 9% did not report their remuneration model.

Most chiropractors reported that 26–50% of their patients presented with acute conditions (39%) and, similarly, 26–50% with chronic conditions (39%). Initial evaluations typically lasted 46–60 min (57%), while treatment sessions most often lasted 16–30 min (57%). Chiropractors reported working alongside a mean of 3.1 family physicians (SD = 3.1) in their clinical settings, the most frequent healthcare provider type present, yet the median percentage of patient referrals originating from physicians was only 7.5% (IQR = 17.5%).

The physician sample (*n* = 19) was predominantly female (90%) with varied experience levels and practice settings, including private practice (58%), hospital-based environments (47%), and multidisciplinary clinics (42%). Most practised general family medicine (74%). For 84% of physicians, patients with musculoskeletal complaints constituted up to half of their patient populations (Table 1).

**Table 1:** Physicians characteristics, sources of information on chiropractic and referral practices (*N* = 19)

Variable		*n*	%
Years in practice			
< 5 years		2	10.5%
5 to 10 years		7	36.8%
11 to 20 years		3	15.8%
> 20 years		7	36.8%
Sources of information on chiropractic^1^			
Personal treatment experience		9	47.4%
Family and friends		8	42.1%
Research literature		2	10.5%
Media		1	5.3%
Medical school		3	15.8%
Patient feedback		15	78.9%
Relationship with a specific chiropractor		12	63.2%
Other		1	5.3%
Number of patients referred for chiropractic care per year			
None		3	15.8%
1 to 10		5	26.3%
11 to 25		8	42.1%
26 to 50		1	5.3%
> 50		2	10.5%
Knowledge of chiropractic			
Little knowledge		9	47.4%
Moderate knowledge		6	31.6%
Very extensive knowledge		4	21.1%
Personally received chiropractic care?			
Yes		13	68.4%
No		6	31.6%
Information about chiropractic during your initial medical training			
Yes, and the information was generally favorable		1	5.3%
Yes, and the information was generally neutral		2	10.5%
Yes, and the information was generally unfavorable		4	21.1%
No		12	63.2%
Should information about chiropractic be presented during initial medical training?			
Yes, definitely		14	73.7%
Possibly		3	15.8%
Uncertain		2	10.5%
Probably not / No definitely not		0	0.0%
Are you comfortable discussing chiropractic care with your patients?			
Yes, definitely		6	31.6%
Somewhat		7	36.8%
Uncertain		4	21.1%
Probably not		1	5.3%
No, definitely not		1	5.3%
Are you interested in learning more about chiropractic?			
Yes		14	73.7%
No		1	5.3%
Uncertain		4	21.1%
When did your opinion on chiropractic primarily develop?			
Before medical school		3	15.8%
During medical school		1	5.3%
After medical school		14	73.7%
Missing		1	5.3%
Should chiropractic care be available in a multidisciplinary environment?			
Yes		13	68.4%
No		1	5.3%
Uncertain		5	26.3%
Should chiropractic care be covered, in whole or in part, by public/government funds?			
Yes		10	52.6%
No		1	5.3%
Uncertain		8	42.1%
Does the diversity of approaches within the chiropractic profession present a barrier to greater collaboration with physicians?			
Yes		7	36.8%
No		4	21.1%
Uncertain		8	42.1%
Do you perceive chiropractors as primary care professionals?			
Yes, definitely		11	57.9%
Somewhat		4	21.1%
Uncertain		3	15.8%
Probably not		1	5.3%
No, definitely not		0	0.0%
Would you like to receive a consultation note from a chiropractor who has seen one of your patients?			
Yes		19	100.0%
No		0	0.0%
Uncertain		0	0.0%
Have patients been referred to you by chiropractors so that you could refer them for imaging examinations?			
Yes		14	73.7%
No		5	26.3%
In your opinion, are adverse effects associated with chiropractic care frequent:			
No		10	52.6%
Uncertain		5	26.3%
Yes		0	0.0%
Yes, but serious events are rare		4	21.1%
In your opinion, what is the level of education required to obtain a license to practice chiropractic?			
College technical degree		0	0.0%
Bachelor’s degree		1	5.3%
First cycle professional doctorate		14	73.7%
Master’s degree		0	0.0%
Doctorate (PhD)		4	21.1%
Where do you think chiropractors should be integrated within the healthcare system? ^1^			
Specialized clinics		4	21.1%
Outpatient clinic in a hospital setting		7	36.8%
Emergency		3	15.8%
Family medicine group/medical clinic.		16	84.2%
Community Health and Social Services Center (CLSC)		3	15.8%
Long-Term Care Facility (CHSLD).		2	10.5%
Readaptation center		7	36.8%
The chiropractors do not have a place within the healthcare system.		0	0.0%
Other		3	15.8%

^1^ Participants could select more than one answer

#### Interprofessional collaboration patterns

Chiropractors spent a median of 60 min weekly (IQR = 41 min) interacting with other healthcare providers, concerning an average of 24% (SD = 20%) of their patients. Informal interactions were most common: 44% of chiropractors reported at least one unplanned, in-person discussion weekly, and 39% reported weekly verbal messages via patients. Formal collaborative activities were infrequent: 48% never scheduled collaborative meetings and 57% never conducted joint evaluations (Table 2).

**Table 2:** Chiropractors’frequency, method and satisfaction with interprofessional collaboration (N=23)

Level of satisfaction	Completely satisfied	Somewhat satisfied	Neither satisfied nor dissatisfied	Somewhat dissatisfied	Completely dissatisfied	Not applicable
Family physicians	5 (21.7%)	**10 (43.5%)**	4 (17.4%)	1 (4.3%)	0 (0.0%)	1 (4.3%)
Specialist physicians	2 (8.7%)	4 (17.4%)	**5 (21.7%)**	4 (17.4%)	1 (4.3%)	5 (21.7%)
Other chiropractors	**8 (34.8%)**	7 (30.4%)	1 (4.3%)	1 (4.3%)	0 (0.0%)	4 (17.4%)
Other healthcare providers (e.g., physiotherapist, occupational therapist, kinesiologist)?	**10 (43.5%)**	8 (34.8%)	2 (8.7%)	0 (0.0%)	0 (0.0%)	1 (4.3%)

Interaction methods	Daily	Weekly	Monthly	Occasionally	Never
Phone discussions	0 (0.0%)	2 (8.7%)	7 (30.4%)	**9 (39.1%)**	4 (17.4%)
Email	2 (8.7%)	4 (17.4%)	**6 (26.1%)**	5 (21.7%)	4 (17.4%)
Letter/form transmitted via patient	0 (0.0%)	7 (30.4%)	**10 (43.5%)**	4 (17.4%)	1 (4.3%)
Letter/form sent by mail or fax	0 (0.0%)	3 (13.0%)	7 (30.4%)	**10 (43.5%)**	2 (8.7%)
Verbal messages via patient	1 (4.3%)	**9 (39.1%)**	6 (26.1%)	2 (8.7%)	4 (17.4%)
In-person unplanned discussions (e.g., hallway)	2 (8.7%)	**10 (43.5%)**	3 (13.0%)	4 (17.4%)	3 (13.0%)
Scheduled in-person meetings	0 (0.0%)	1 (4.3%)	4 (17.4%)	5 (21.7%)	**11 (47.8%)**
Joint patient evaluation/intervention	0 (0.0%)	0 (0.0%)	3 (13.0%)	6 (26.1%)	**13 (56.5%)**

Frequency of interactions with providers	Daily	Weekly	Monthly	Occasionally	Never
Family physicians	4 (17.4%)	7 (30.4%)	**8 (34.8%)**	3 (13.0%)	0 (0.0%)
Sports medicine physicians	2 (8.7%)	4 (17.4%)	2 (8.7%)	4 (17.4%)	**10 (43.5%)**
Neurologists	0 (0.0%)	0 (0.0%)	1 (4.3%)	7 (30.4%)	**12 (52.2%)**
Orthopedists	0 (0.0%)	1 (4.3%)	2 (8.7%)	9 (39.1%)	**10 (43.5%)**
Rheumatologists	1 (4.3%)	0 (0.0%)	1 (4.3%)	6 (26.1%)	**12 (52.2%)**

Mode: Bold

Family physicians were chiropractors’ most frequent healthcare contacts with 35% of chiropractors reporting at least one monthly contact and 30% reporting at least one weekly contact (Table 3). Interactions with specialists were less frequent, with over 40% reporting never interacting with neurologists, rheumatologists, or orthopedists (Table 6). Most chiropractors (70%) reported no scheduled clinical case discussions with other providers. Resources for collaboration included professional email (70%), fax (74%), and electronic health records (52%).

**Table 3:** Chiropractors’ perception of interprofessional Collaboration and values importance (n = 23)

Domain	Mean ± SD
*Perceptions for each of the questions, (0* = *not at all, 10* = *completely)*	
To what extent did you take into consideration all the needs of your patients, i.e., physical, psychological, and social needs?	8.3 ± 1.3
To what extent did you take into consideration the data collected by other providers?	7.9 ± 1.9
To what extent did you refer your patients to other providers?	7.5 ± 2.1
To what extent were your professional relationships with different providers equal?	7.7 ± 2.1
To what extent did you cooperate with other providers to ensure the follow-up of shared patients?	7.4 ± 2.3
To what extent did you exchange information with other providers concerning shared patients?	7.3 ± 2.4
To what extent did you seek advice or opinions from other providers?	7.0 ± 2.4
To what extent did you tolerate the existence of gray areas in sharing responsibilities with other providers?	6.0 ± 2.6
To what extent were your clinical activities with patients coordinated with the clinical activities of other providers with the same patients?	6.2 ± 2.9
To what extent did you develop common intervention plans with other providers?	5.0 ± 2.9
To what extent was the sharing of responsibilities between you and other providers pre-established?	4.8 ± 3.1
To what extent did you make efforts to avoid creating conflicts regarding the sharing of tasks and responsibilities with other providers?	4.6 ± 3.7
*Values importance within the clinic (0* = *no importance, 10* = *highest importance)*	
Respect, courtesy, and confidentiality	9.6 ± 0.8
Patients' physical health	9.4 ± 0.8
Patients' psychological and social health	8.7 ± 1.3
Prevention and health promotion	8.6 ± 2.0
Interactions between chiropractors and other providers	8.5 ± 2.0
Evidence-based practice	7.8 ± 2.0
Financial profitability	6.8 ± 2.3

Among physicians, 68% referred patients to chiropractors either monthly (42%) or weekly (26%), driven primarily by established relationships with specific chiropractors (53%), patient request (47%), and clinical evidence supporting chiropractic care for certain conditions (37%). The majority (79%) reported chiropractic services available at or near their practice location (Table 1).

#### Perceptions and attitudes

On a 0–10 scale (0 = not at all, 10 = completely; higher scores indicating greater enactment of that collaborative behaviour over the past 12 months), chiropractors rated the following dimensions highest in terms of the importance of interprofessional collaboration: consideration of patients’ comprehensive needs (M = 8.3, SD = 1.3), incorporation of information from other providers (M = 7.9, SD = 1.9), and equality in professional relationships (M = 7.7, SD = 2.1). Lower ratings were observed for development of common intervention plans (M = 5.0, SD = 2.9) and pre-established responsibility sharing (M = 4.8, SD = 3.1). With respect to interprofessional values, Chiropractors rated respect and confidentiality highest (M = 9.6/10, SD = 0.8), followed by patients’ physical health (M = 9.4/10, SD = 0.8) and patients’ psychological/social health (M = 8.7/10, SD = 1.3) (Table 3).

**Table 4:** Physicians’ attitude towards chiropractic (N=19)

	Strongly agree, n (%)	Agree, n (%)	Undecided, n (%)	Disagree, n (%)	Strongly disagree, n (%)
Chiropractors promote unnecessary treatment plans	0 (0.0%)	2 (10.5%)	6 (31.6%)	**7 (36.8%)**	**4 (21.1%)**
Chiropractors provide effective therapy for some musculoskeletal conditions	**8 (42.1%)**	**9 (47.4%)**	1 (5.3%)	0 (0.0%)	1 (5.3%)
Chiropractors make excessive use of radiographic imaging	1 (5.3%)	1 (5.3%)	6 (31.6%)	**8 (42.1%)**	**5 (15.8%)**
Chiropractors provide a patient centered approach	**6 (31.6%)**	**11 (57.9%)**	2 (10.5%)	0 (0.0%)	0 (0.0%)
I have to spend time correcting erroneous information patients have received from chiropractors	0 (0.0%)	1 (5.3%)	3 (15.8%)	**10 (52.6%)**	**5 (26.3%)**
Chiropractic manipulation of the neck is generally a safe therapy	**2 (10.5%)**	**5 (26.3%)**	6 (31.6%)	5 (26.3%)	1 (5.3%)
Chiropractors can provide effective therapy for some non- musculoskeletal conditions	**3 (15.8%)**	**7 (36.8%)**	5 (26.3%)	3 (15.8%)	1 (5.3%)
Family physicians may risk professional liability if they refer a patient to a chiropractor	0 (0.0%)	6 (31.6%)	2 (10.5%)	**4 (21.1%)**	**7 (36.8%)**
Chiropractors can reduce patient overload for family physicians with respect to patients with musculoskeletal complaints	**7 (36.8%)**	**11 (57.9%)**	1 (5.3%)	0 (0.0%)	0 (0.0%)
Chiropractors provide patients with misinformation regarding vaccination	0 (0.0%)	2 (10.5%)	5 (26.3%)	**4 (21.1%)**	**8 (42.1%)**
Chiropractic provides effective therapy for post-surgical rehabilitation	1 (5.3%)	8 (42.1%)	**10 (52.6%)**	0 (0.0%)	0 (0.0%)
Chiropractors lack sufficient clinical training	0 (0.0%)	0 (0.0%)	9 (47.4%)	**5 (26.3%)**	**5 (26.3%)**
Chiropractic care is a useful supplement to conventional medicine	**9 (47.4%)**	**8 (42.1%)**	1 (5.3%)	1 (5.3%)	0 (0.0%)
Chiropractors engage in overly aggressive marketing	0 (0.0%)	2 (10.5%)	4 (21.1%)	**8 (47.4%)**	**4 (21.1%)**
Chiropractic includes ideas and methods from which conventional medicine could benefit	**2 (10.5%)**	**13 (68.4%)**	4 (21.1%)	0 (0.0%)	0 (0.0%)
The results of chiropractic manipulation are due to the placebo effect	0 (0.0%)	0 (0.0%)	3 (15.8%)	**10 (52.6%)**	**6 (31.6%)**
Chiropractors treat in accordance with evidence-based practices	**5 (26.3%)**	**10 (52.6%)**	4 (21.1%)	0 (0.0%)	0 (0.0%)
Chiropractic has no role in the routine care of my patients	0 (0.0%)	1 (5.3%)	3 (15.8%)	**7 (36.8%)**	**8 (42.1%)**
Chiropractic breeds dependency in patients on short-term symptomatic relief	0 (0.0%)	2 (10.5%)	1 (5.3%)	**8 (42.1%)**	**8 (42.1%)**
	**Very good (%)**	**Good (%)**	**Undecided (%)**	**Poor (%)**	**Very poor (%)**
General impression about chiropractic	**7 (36.8%)**	**7 (36.8%)**	5 (26.3%)	0 (0.0%)	0 (0.0%)

CAQ: mean = 57.2; Standard Deviation = 11.2;

Bold: most endorsed cluster for each item Strongly agree + Agree; Undecided; Disagree + Strongly disagree

In terms of satisfaction with interprofessional interactions, 78% of chiropractors reported being completely or somewhat satisfied with their collaboration with allied health professionals (e.g., physiotherapists, occupational therapists, kinesiologists) and 65% were satisfied with interactions with family physicians; however, only 26% were satisfied with interactions involving specialist physicians (Table 2).

Most physicians surveyed (74%) had an overall positive impression of chiropractic care and agreed that chiropractors provide effective therapy for musculoskeletal conditions (90%), employ patient-centred approaches (90%), and can reduce physician overload for musculoskeletal complaints (95%) (Table 4). However, opinions were more varied regarding the safety of neck manipulations (37% agreed, 32% undecided, 32% disagreed). Concerns about the risk of professional liability from patient referrals to a chiropractor similarly varied with 32% agreeing there is increased risk, and 58% disagreeing (Table 4).

**Table 5:** Physicians’ perception of their collaboration with chiropractors (N=19)

	Strongly agree, n (%)	Agree, n (%)	Undecided, n (%)	Disagree, n (%)	Strongly disagree, n (%)
You are satisfied with the collaboration with chiropractors in your practice environment	**9 (47.4%)**	**5 (26.3%)**	5 (26.3%)	0 (0.0%)	0 (0.0%)
The collaboration with chiropractors requires a significant time investment	0 (0.0%)	2 (10.5%)	3 (15.8%)	**12 (63.2%)**	**2 (10.5%)**
Chiropractors bring relevant suggestions for patient care	**5 (26.3%)**	**11 (57.9%)**	2 (10.5%)	1 (5.3%)	0 (0.0%)
Your collaboration with chiropractors has allowed you to develop your knowledge regarding chiropractic care	**6 (31.6%)**	**6 (31.6%)**	4 (21.1%)	3 (15.8%)	0 (0.0%)
In the future, you desire to continue collaboration with chiropractors in the same way or in a similar manner	**8 (42.1%)**	**7 (36.8%)**	4 (21.1%)	0 (0.0%)	0 (0.0%)

The collaboration and my interactions with chiropractors:	Strongly agree, n (%)	Agree, n (%)	Undecided, n (%)	Disagree, n (%)	Strongly disagree, n (%)
Enhance the health condition of my patients	**7 (36.8%)**	**9 (47.4%)**	3 (15.8%)	0 (0.0%)	0 (0.0%)
Help to clarify the diagnosis of my patients' presenting complaints	**5 (26.3%)**	**9 (47.4%)**	4 (21.1%)	1 (5.3%)	0 (0.0%)
Enhance patient satisfaction	**6 (31.6%)**	**10 (52.6%)**	3 (15.8%)	0 (0.0%)	0 (0.0%)
Aid in determining relevant diagnostic tests for my patients	2 (10.5%)	1 (5.3%)	**13 (68.4%)**	2 (10.5%)	1 (5.3%)
Aid in determining the patient's care trajectory (other healthcare providers, specialists, or services)	2 (10.5%)	4 (21.1%)	**10 (52.6%)**	3 (15.8%)	0 (0.0%)
Decrease my workload	**4 (21.1%)**	**6 (31.6%)**	5 (26.3%)	3 (15.8%)	1 (5.3%)
Enhance the quality of services my patients receive	**6 (31.6%)**	**10 (52.6%)**	3 (15.8%)	0 (0.0%)	0 (0.0%)

Bold: most endorsed cluster for each item Strongly agree + Agree; Undecided; Disagree + Strongly disagree

Most physicians (74%) reported being satisfied with their collaboration with chiropractors, and an equal proportion (74%) disagreed that collaboration required a significant time investment. Most physicians agreed that collaboration enhanced patient satisfaction (84%), improved service quality (84%), and positively impacted patients’ health conditions (84%) (Table 5).

**Table 6:** Chiropractors’ Perceived Facilitators, Barriers and benefits to Interprofessional Collaboration (n=23)

	Major Facilitators	Minor Facilitators	Neither facilitator nor barrier	Minor Barriers	Major Barriers	Not applicable
Personally knowing other providers	**19 (82.6%)**	0 (0.0%)	1 (4.3%)	0 (0.0%)	0 (0.0%)	0 (0.0%)
Presence of other providers in proximity	**14 (60.9%)**	3 (13.0%)	2 (8.7%)	0 (0.0%)	0 (0.0%)	1 (4.3%)
Patient condition complexity	**6 (26.1%)**	4 (17.4%)	4 (17.4%)	5 (21.7%)	1 (4.3%)	0 (0.0%)
Chiropractor remuneration model	2 (8.7%)	1 (4.3%)	**11 (47.8%)**	4 (17.4%)	0 (0.0%)	2 (8.7%)
Chiropractor workload	0 (0.0%)	2 (8.7%)	7 (30.4%)	**8 (34.6%)**	3 (13.0%)	0 (0.0%)
Other providers' workload	0 (0.0%)	3 (13.0%)	**6 (26.1%)**	3 (13.0%)	**6 (26.1%)**	2 (8.7%)
Patients' precarious financial situation	2 (8.7%)	3 (13.0%)	3 (13.0%)	**5 (21.7%)**	**5 (21.7%)**	2 (8.7%)

Perceived benefits of collaboration	Strongly agree	Agree	Neither Agree nor Disagree	Disagree	Strongly Disagree
Increased patient satisfaction	**17 (73.9%)**	3 (13.0%)	1 (4.3%)	0 (0.0%)	0 (0.0%)
Helped promote chiropractic to other providers	**14 (60.9%)**	5 (21.5%)	1 (4.3%)	1 (4.3%)	0 (0.0%)
Improved patients' clinical condition	**10 (43.5%)**	9 (39.1%)	2 (8.7%)	0 (0.0%)	0 (0.0%)
Better addressed patients' biopsychosocial needs	**11 (47.8%)**	7 (30.4%)	1 (4.3%)	1 (4.3%)	0 (0.0%)
Increased referrals to chiropractic	**9 (39.1%)**	6 (26.1%)	5 (21.5%)	1 (4.3%)	0 (0.0%)
Increased job satisfaction	7 (30.4%)	**10 (43.5%)**	3 (13.0%)	1 (4.3%)	0 (0.0%)

Mode: Bold

#### Facilitators, barriers, and perceived benefits

The strongest facilitators of collaboration identified by chiropractors were personally knowing other providers (83%), provider proximity (74%), and patient condition complexity (44%). The primary barriers included chiropractor’s workload (48%), other providers’ workload (39%), and patients’ financial situations (43%) (Table 6).

Chiropractors overwhelmingly reported positive impacts of collaboration, including increased patient satisfaction (87%), greater promotion of chiropractic care to other providers (83%), improved clinical outcomes (83%), and better handling of patients’ biopsychosocial needs (78%) (Table 6).

Most physicians (68%) believed chiropractic care should be available in multidisciplinary environments, with 53% supporting partial public funding. The majority (84%) selected family medicine groups as the most appropriate integration setting for chiropractic care (Table 1). When asked about obstacles to integration in the survey’s open-ended questions, physicians cited out-of-pocket costs to patients, lack of knowledge among physicians about chiropractic care, practice variability among chiropractors, and system integration issues.

### Qualitative results

This section presents the results of the thematic analysis of qualitative data collected from seven chiropractors (focus group) and five physicians (individual semi-structured interviews) regarding their experiences of interprofessional collaboration. Three overarching themes emerged from the analysis: (1) Perception, (2) Organization of care and services, and (3) Communication. Each theme encompasses several subthemes. Participants also proposed various improvement strategies that will be presented in the final part of this section. Table 7 presents each theme, subtheme, and supporting quotations labelled by participant (DC1 to DC7 = chiropractor 1 to 7; MD1 to MD5 = physician 1 to 5). The qualitative and quantitative samples components drew on the same eligible population: all co-located chiropractors and physicians were invited to complete the survey and to take part in the focus group or interviews. The samples overlap but are not nested, as some qualitative participants did not complete the survey.

**Table 7:** Results and supporting quotations from the qualitative analysis

Theme	Subtheme	Quotation
1. Perception	1.1 Mutual perception of competencies and roles	“For disorders of the spine, often, it's the group that will have the best expertise.” MD3
		“In terms of musculoskeletal, I find that chiropractors are really better trained than we are.” MD1
		“The chiropractor sees more globally than the physiotherapist, that's my personal opinion. I find that the global vision of the patient is much greater with a chiropractor.” MD5
		"In all honesty, I don't really know much, I know you have long studies, I know it's a doctorate […] but I haven't been exposed to chiropractic." MD2
		"The history or all the prejudices that physicians have towards the profession […] it's not all for nothing that they have apprehensions." DC1
		"The preconceived ideas are actually not so bad according to the studies. So I think we always hear the worst, but it's not so general." DC5
	1.2 Perceptual barriers	“During my training in the Locomotor phase in musculoskeletal, we were told to send to physiotherapist, send to osteo for certain specific pathologies and not to send to chiropractors.” MD2
		"In medical school, but there's no chiropractic in hospitals, so there's no interaction during all of medical training." MD3
		“I think what's harmful is more a history. I think there was a war between chiropractors and physicians […] this image that remains, and I think that unfortunately, there are still a few chiropractors [focussing on high patient volume] so I think that this image remains a bit and harms the profession a bit.” MD3
		"It's a diversity of practices also, the different approaches, different techniques. Sometimes, physicians will say: ‘One chiropractor did that [problematic thing], so they all do that' or 'If they did that once, they will always do that’. It can also complicate communication, referral." DC3
		"For more articular pathologies, soft tissues or others, it seems to be more chiropractor-dependent." MD3
	1.3 Perceptual facilitators	“The physicians around me who refer to chiropractors […] are all physicians who have consulted themselves.” MD2
		“One of the things I find important when you have the chance to work in the same office is the continuing education we can do with them. […] education is really done daily.” DC4
2. Organisation of Care and Services	2.1 Benefits of collaboration – for patients, professionals and the healthcare system	“The patient will feel more supported, will feel more surrounded, and will feel supported in everything.” DC4
		“It allows us to take care of our patients a bit faster […] these are families who don't have money, so it makes a really big difference.” MD4
		"The positive side is that they take less medication." MD5
		“It allows me to have better access to patients who really need to see me. I think it distributes patients to the right place to get the right treatments.” MD5
		"At the beginning of practice, when you're trying to build a practice, having referrals, it's certainly easier when it's the physician who refers often." DC6
		"It really decongests the system a lot. I find, and even for follow-up it also decongests their physician." (MD1)
		"It's appointments with specialists. So we don't congest the list even more, it's unnecessary delays that are much shorter, it's money saved for everyone." DC3
	2.2 Structural barriers	“I have lots of patients that I'd like to send to see a chiropractor. But there's a financial problem.” MD5
		"The fees for chiropractic consultations. I think there are many for whom it's financial means […] unfortunately they don't have the means." MD1
		“The fact that we're not in hospitals means we have much less contact with physicians than physiotherapists can have because they're in virtually all hospitals.” DC6
		"I think integration into the healthcare system would be logical. And we see that it's perhaps one of the professions that isn't integrated compared to others." MD3
		"All the paperwork, […] I'm quite passive-aggressive in my comments now. I write 'see report and suggestions from the chiropractor' and I sign the work stoppage." MD5
		"In fact, I think that the work stoppage that is really related to musculoskeletal is something that the chiropractor can manage." MD5
	2.3 Structural facilitators	“Same building. […] The Physio clinic, and a pharmacy, it was easier.” MD5
		"Being in the same clinic as if we eat at the same place also means there's a bit of exchange that happens." DC7
		“It's perhaps just that the service corridors are really clear for all healthcare professionals.” MD5
		"When it's more spine, cervical, thoracic lumbar, I'll be more inclined to refer to chiro. If it's more rehabilitation of injuries, exercise program, I'll be more inclined to refer to physiotherapy." MD3
3. Communication	3.1 Communication modalities – direct verbal, written, formal meetings	“We have direct interactions, we discuss cases directly at the office level.” MD3
		"I'll go see them between 2 patients in their office." DC3
		"I'm quite telephone-oriented. It avoids the hassle of 'I'm not sure I understand', and the way you write your physical exams, sometimes I don't know the abbreviations." MD5
		“For chiropractors who are not at the clinic, they send me a note when the patient arrives, then I'll return a note to them.” MD3
		"We operate with notes really, small handwritten notes. The patient arrives with these things, and it's the patient who is the messenger." DC5
		"We hold interdisciplinary meetings every 2 weeks to discuss cases. […] with physicians, we schedule it more like every 2–3 months." DC2
	3.2 Communication barriers	“If we were paid like other professionals, with time that we can take more, I think that would allow these communications.” MD2
		"I find it hard to take the time to write the letter […] more complex cases of CNESST of SAAQ, […] it's worth it, to pick up the phone like I call a physician colleague, just so we're sure we understand each other." MD4
		“Yeah, no, it's very closed […] I had Miles (electronic records) too and it didn't even work together.” MD5
		"Having the same records indeed, yes, it's good. Already the Quebec Health Record, I think it's a step in the right direction." DC3
		"It's much more me who takes the steps, who sends the emails, who sends the communication to the physicians." DC7
		"I think it's easier. I think when a physician calls a chiropractor, it's easy. I think when a chiropractor wants to talk to a physician, it's more difficult." MD1
	3.3 Communication facilitators	“I know them, it also helps. I trust them and their judgment.” MD5
		"I like to have the written record too. It's especially for my files, especially CNESST cases, SAAQ or work stoppage." MD5
		"It's sure that I try not to write for nothing, because they receive an astronomical quantity of documents, and laboratory reports, there's a lot to manage." DC6

#### Characteristics of participants

Chiropractors (*n* = 7) practised in various collaborative contexts with physicians, including multidisciplinary clinics with an on-site medical presence (*n* = 4), clinics adjacent to Family Medicine Groups (*n* = 2), and an independent practice with external collaborations (*n* = 1). Years in practice ranged from 2 to over 15 years.

Physicians (*n* = 5) represented different specialties and practice contexts: one emergency physician, three family physicians (including one practising exclusively musculoskeletal medicine outside the public healthcare system), and one pediatrician working in social pediatrics. Two of the participating physicians were in a personal relationship with a chiropractor.

#### Theme 1: Perception

The “Perception” theme encompasses how the two professional groups view each other’s competencies, roles, and value in the healthcare system, as well as how these perceptions influence collaboration.

#### Subtheme 1.1: Mutual perception of competencies and roles

As shown in Table 7, many physicians acknowledged chiropractors’ specialized skill in musculoskeletal care, particularly for spinal conditions, and perceived their training as complementary to their own. As one physician explained, this reflected an exchange of complementary competencies and areas of expertise between the two professions (Table 7). Many physicians perceived chiropractors as having a more holistic approach to patient care compared to other professionals. Some physicians admitted minimal experience with chiropractic practice during their medical education and clinical training. Chiropractors themselves were acutely aware of this knowledge gap and the lingering effects of historical interprofessional tensions.

#### Subtheme 1.2: Perceptual barriers

Participants described three main perception-related barriers that undermine effective collaboration: educational bias, historical tensions, and variable practice approaches. First, many physicians reported that their formal training actively de-emphasized chiropractic care; chiropractic was notably absent from hospital-based rotations, fostering a professional habit of overlooking it as a valid treatment option. Second, a legacy of interprofessional conflict continues to shape current attitudes. Chiropractors noted these challenges to collaboration:

*“There are still preconceived ideas. Some want nothing to do with it*,* or have a lot of apprehension*,* and will still refer to physiotherapy for cases we could treat.”* (DC3). Finally, physicians perceived the heterogeneity of chiropractic practice as confusing, which may lead to referral hesitancy. Initial uncertainty about scope of practice was echoed by physicians as well: *“We were a bit ambivalent*,* asking ourselves: ‘What will the contribution be?’ […] we don’t know what the indications are for referring to a chiropractor.”* (MD4).

#### Subtheme 1.3: Perceptual facilitators

Several factors helped overcome negative preconceptions. Physicians who had personal experience with chiropractic care tended to hold more favourable attitudes. Direct professional exposure, particularly working alongside chiropractors in the same clinical setting, further enhanced mutual respect. As one physician described, perceptions shifted through direct professional contact: *“It was really her who approached us*,* who took the time to introduce herself and clearly explain her scope of practice.”* (MD4). When chiropractors demonstrated their competencies, physicians reported that initial concerns about safety and professional boundaries were progressively resolved.

*Perception-focused strategies* Embedding chiropractic content within undergraduate and postgraduate medical curricula would address educational gaps and counter long-standing stereotypes. Increasing chiropractor representation at medical conferences and joint continuing-education events could foster mutual familiarity and trust. Establishing collaborative research initiatives offered another avenue to build shared evidence and demonstrate the value of integrated care.

“*Being present in multidisciplinary settings at school*,* at the level of studies*,* from both sides*,* both at the physician and chiro level*.” (DC5).

“*I think that putting it forward in continuing medical education can help having chiropractors who present*.” (MD3).

#### Theme 2: Organization of care and services

The “organization of care and services” theme addresses the structural aspects of collaboration, including care pathways, roles in patient management, and system integration (see Table 7).

#### Subtheme 2.1: Benefits of collaborative organization

Participants identified clear benefits of collaboration at the patient, professional, and system levels. At the patient level, collaboration improved the comprehensiveness and timeliness of care. One chiropractor illustrated how co-management could add clinical value, describing the everyday, informal continuing education that co-location makes possible and that helps clarify which conditions chiropractors can manage rather than refer (Table 7). At the system level, joint management of musculoskeletal conditions reduced unnecessary testing:

*“We can avoid unnecessary tests or better target the ones that are needed. If we know how to talk between professionals*,* sometimes you see a whole battery of tests ordered that aren’t necessarily justified.”* (DC1). Physicians confirmed this perspective: *“For the musculoskeletal system*,* I find it really enhances patient management […] she would note red flags but also small particularities that warranted further investigation.”* (MD4).

#### Subtheme 2.2: Structural barriers

Participants identified three key structural obstacles. First, out-of-pocket costs for chiropractic care were repeatedly cited as a major impediment, particularly for vulnerable populations. Second, the exclusion of chiropractors from public healthcare settings was viewed as a systemic barrier that limits opportunities for direct interaction: *“If we could refer more easily*,* if there wasn’t the issue of reference toward specialists*,* rheumatologists or orthopedists*,* without going through a family physician.”* (DC1). Without formal integration, chiropractors have limited visibility within medical networks. Finally, administrative constraints and unclear documentation protocols were described as time-consuming.

#### Subtheme 2.3: Facilitators of care organization

Physical proximity emerged as a central facilitator, enabling spontaneous case discussions and informal knowledge exchange. Formal interprofessional meeting structures were also valued when available: *“We are truly in collaboration with the Family Medicine Group located just one floor up […] two to three times a year*,* we gather about fifty professionals in a room to discuss collaboration.”* (DC2). Established and well-defined referral pathways were viewed as essential for improving care coordination.

*Organization of care strategies* Integrating chiropractors into public healthcare settings, such as family medicine groups, would improve accessibility and streamline referral pathways. Enabling chiropractors to complete and sign the work-absence certificates and other documentation required by workers’ compensation and automobile insurance boards for musculoskeletal conditions could reduce physician workload and formalize collaborative roles. Finally, clear referral pathways and service corridors as well as public funding models for chiropractic care would reduce financial barriers and embed chiropractors within established care networks.

“*It’s certainly that having chiropractors*,* a bit like physiotherapists in FMGs*,* in family medicine clinics who can see patients at the expense*,* a bit of the clinic*,* patients who don’t have insurance*,* that could be really positive*.” (MD1).

”*Yes*,* enormously*,* it frees us up. I have been counting the number of appointments I see for nothing in a week*,* and there are a lot of them.*” (MD5).

#### Theme 3. Communication

The third theme addressed methods, content, frequency, and quality of interprofessional exchanges, as well as barriers and facilitators to effective communication between chiropractors and physicians (see Table 7).

#### Subtheme 3.1 Communication modalities

Participants reported employing a mix of verbal, written, and formal meeting formats. Direct verbal communication, whether face-to-face hallway conversations or quick telephone calls, was cited as the most effective modality: *“You can speak directly with physicians*,* know what they’re doing […] it can happen by phone*,* it can happen simply in the corridor—it’s really quick.”* (DC2). Written communication was used primarily when professionals were not co-located, with patients sometimes carrying handwritten notes between providers and effectively serving as the messenger (Table 7). Shared electronic records, where available, were seen as a significant advance: *“Working in the same files*,* we use the same software […] if the patient agrees*,* we have access to all visits*,* the full history*,* in much more detail than simply what the patient tells us.”* (DC7).

#### Subtheme 3.2: Communication barriers

Participants identified three principal obstacles. First, heavy workloads and billing structures leave insufficient time for communication. Second, siloed information systems created barriers to seamless information exchange. Third, and most notably, an imbalance in communication initiation was identified, with chiropractors describing themselves as the ones who most often initiate contact, send the emails, and follow up with physicians (Table 7). This asymmetry places a disproportionate burden on chiropractors to maintain interprofessional dialogue.

#### Subtheme 3.3: Communication facilitators

Two key enablers emerged. First, established relationships fostered mutual trust and eased clinical information exchange. One chiropractor noted that physicians who had themselves been patients subsequently became active referrers: *“Physicians who came as patients*,* and who after that started referring.”* (DC5). Second, concise and targeted communication was valued by both groups, allowing each profession to integrate external clinical input efficiently. Physicians noted that when communication was clear and professionally framed, it reduced hesitation and smoothed collaborative workflows.

*Communication strategies* Allocating dedicated time for interprofessional case discussions would help providers engage in meaningful dialogue. Developing or adopting shared information systems or improving interoperability as well as implementing standardized referral templates would minimize documentation burdens and prevent information siloing. Promoting direct, bidirectional communication channels (e.g., dedicated secure messaging lines) would balance the onus of initiation and reinforce equitable professional relationships.

“*If [all] physicians could have one hour in the afternoon*,* with no patients*,* that [would be] open for communications with other professionals.*” (MD2).

”*At the level of referrals*,* often*,* it’s going a bit in both directions. If we send patients with referral notes*,* explanatory notes*,* explaining a bit the plan. But I think that can help to get patients returned*,* these patients and sometimes other patients*,* to create a bit of a connection.*” (MD3).

### Integration of quantitative and qualitative findings

Reading the two datasets together produced insights that neither strand yielded alone (Table 8). First, it resolved an apparent survey contradiction: physicians endorsed chiropractic effectiveness almost unanimously (90%) yet were evenly split on cervical-manipulation safety (37% agreed it is safe). The interviews traced this ambivalence not to doubts about the evidence but to concerns inherited from training and professional history, and showed it easing after direct, co-located evidence-sharing; integration thus reframes the 37% as a remediable information deficit rather than a fixed objection.

**Table 8:** Joint display integrating quantitative (survey) and qualitative (focus group and interview) findings, organized by meta-inference

Integrated dimension and fit	Quantitative findings (surveys)	Qualitative findings (themes and representative quotations)	Meta-inference (integrated interpretation)
1. Perceived effectiveness versus safety and liability ambivalence *Fit: expansion*	Physicians rated chiropractic effective for musculoskeletal conditions (90%), patient-centred (90%), and able to reduce physician overload (95%) Yet ratings were split on the safety of cervical manipulation (37% agreed it is safe, 32% undecided, 32% disagreed) and on increased liability risk from referral (32% agreed, 58% disagreed)	Favourable effectiveness views were grounded in direct clinical experience, whereas safety concern was traced to training and professional history rather than to current evidence: *“There is still a slide at the medical school in Montréal saying the patient presents with a stroke following their chiropractic appointment.” (DC5)* It was reinforced by perceived heterogeneity of practice: *“It is a diversity of practices… if they did that once, they will always do that.” (DC3)* The concern proved reversible through dialogue: two paediatricians’ initial neck-manipulation and professional-responsibility worries eased after the co-located chiropractor “sent plenty of studies on neck manipulations” and clarified indications and scope of practice (MD4)	The qualitative data reframe the split safety ratings as an information deficit inherited from training and professional history, amenable to co-location and direct evidence-sharing, rather than a rejection of evidence physicians already endorse. Neither strand alone yields this: the survey shows the ambivalence; the interviews show it is contingent and remediable
2. High perceived value versus low formalization *Fit: expansion (central integrated finding)*	Physicians reported that collaboration improved patient satisfaction (84%), service quality (84%), and health outcomes (84%); 74% were satisfied with it Formal collaboration was nonetheless rare: 48% never scheduled meetings, 57% never conducted joint evaluations, and 70% held no scheduled case discussions; interactions were mostly informal (44% weekly unplanned in-person; 39% patient-relayed)	Collaboration was sustained by proximity and informal exchange rather than embedded processes: *“I will go see them between two patients in their office.” (DC3)* *“We operate with notes really, small handwritten notes… the patient who is the messenger.” (DC5)* Co-location and clear service corridors were named as the main enablers (MD5)	High perceived value coexists with minimal structure. Read together, the survey “never” responses index reliance on informal, relationship-driven collaboration that is effective but fragile, not an absence of collaboration. This is the study’s central integrated finding and the principal reason the two datasets must be interpreted jointly rather than as separate papers
3. Financial barriers and access equity *Fit: confirmation, enriched and expanded*	Patients’ financial situation was among chiropractors’ primary barriers (43%), consistent with a predominantly fee-for-service model (70%) A majority of physicians (84%) selected Family Medicine Groups as the most appropriate setting for integrating chiropractic care	The barrier was confirmed and specified as an equity concern: *“There is a financial problem… they do not have the means.” (MD5)* *“Some insurance plans cover a better percentage for physiotherapy consultations than for chiropractic.” (MD1)* *“The fact that we are not in hospitals means we have much less contact with physicians.” (DC6)*	Strands converge, and the qualitative data convert an abstract quantitative barrier into a concrete equity mechanism: private out-of-pocket payment, reimbursement that favours physiotherapy, and exclusion from publicly funded settings. The integrated reading points to clinic-funded access within Family Medicine Groups as the participants’ preferred remedy
4. Communication profile and relational asymmetry *Fit: discordance*	Collaboration relied on fax (74%) and patient-relayed messages (39%), with shared records in only about half of settings (52%) Chiropractors’ lowest self-rated collaborative behaviours were development of common intervention plans (M = 5.0) and pre-established responsibility sharing (M = 4.8) These sit against physicians’ self-report of patient-centred practice (90%) and reduced physician overload (95%)	Coordination was frequently offloaded onto patients, and shared software marked the divide between fluid and effortful exchange: *“We operate with… the patient who is the messenger.” (DC5)* Initiation was asymmetric: *“It is much more me who takes the steps, who sends the emails.” (DC7)*	The self-reported patient-centredness and overload reduction sit in tension with a coordination burden shifted onto patients and a relational labour borne mainly by chiropractors. Interpreting the strands together reframes low formalization as a structural imbalance rather than mutual disengagement, and directly motivates protected time and standardized, bidirectional referral templates

Note. Fit categories follow Fetters, Curry and Creswell (2013): confirmation (strands agree), expansion (one strand explains or extends the other), and discordance (strands are in tension). Quantitative percentages and means are drawn from Tables 1, 2, 3, 4, 5, 6; representative quotations correspond to the themes reported in Table 7 (DC1 to DC7 = chiropractor1 to 7; MD1 to MD5 = physicians 1 to 5). Quotations are English translations of French-language data

Second, integration explained the coexistence of high perceived value (84% reported improved care quality; 74% satisfied) with limited collaboration protocols (roughly half to two thirds never held meetings, joint evaluations, or case discussions). The qualitative data showed collaboration sustained by informal, proximity-based exchange rather than embedded processes, indicating reliance that is effective but fragile rather than an absence of collaboration. This value-structure gap is the study’s central integrated finding.

Third, the qualitative accounts converted an abstract financial barrier into a specific equity concern, linking the 43% of chiropractors citing patients’ finances and the 84% favouring the Family Medicine Group model to vulnerable patients who cannot pay and to reimbursement that favours physiotherapy; the preferred remedy was clinic-funded access within Family Medicine Groups.

Finally, the strands diverged in one respect, the clearest instance of discordance: physicians rated their practice as patient-centred (90%) and as reducing physician overload (95%), yet coordination often depended on patients relaying information themselves, and chiropractors initiated most contact (reflected in the lowest collaborative-behaviour ratings: common intervention plans Mean = 5 /10, shared responsibility Mean = 5/10). Read together, low formalization reflects a structural imbalance in relational labour rather than mutual disengagement, and points directly to protected time and standardized, bidirectional referral templates.

## Discussion

Interpreted through the framework proposed by Mior and colleagues for physician-chiropractor collaboration in primary care [7], the three themes identified in this study align coherently with the framework’s core categories. The *Communication* theme maps directly onto Mior’s *communication* category, confirming that both formal and informal exchange modalities, including co-location, shared records, and concise referral tools, remain central determinants of collaborative practice. The *Organization of care and services* theme reflects Mior’s *service delivery* category, with financial barriers, exclusion from public settings, and administrative constraints emerging as persistent structural impediments, consistent with findings from their Ontario-based study conducted fifteen years earlier [7]. The *Perception* theme aligns most closely with Mior’s *practice parameters* category, insofar as physicians’ uncertainty about chiropractic scope of practice, heterogeneity of techniques, and lingering historical tensions continue to create referral hesitancy. Taken together, these convergences suggest that the barriers to physician-chiropractor collaboration identified by Mior and colleagues in 2010 remain unresolved in Quebec’s primary care context, underscoring the need for acting simultaneously on relational, organizational, and structural dimensions.

A central finding of this study is that collaboration in multidisciplinary settings is broadly valued and perceived as clinically beneficial by both professional groups, yet it remains structurally underdeveloped. Both chiropractors and physicians attributed high value to collaboration, reporting improvements in patient satisfaction, clinical outcomes, and care quality. However, actual collaborative practice was characterized by informal, relationship-driven interactions, spontaneous hallway conversations, patient-mediated communication, and referrals driven by personal acquaintance, rather than formalized organizational frameworks. Busse and colleagues have previously documented that most orthopedic surgeons and family physicians perceived chiropractic care as effective for musculoskeletal conditions while the majority refer few to no patients for this type of care [21, 22]. This gap between perceived value and structural reality is consistent with the persistent structural barriers documented by Mior and colleagues [7] in Ontario, and echoes broader evidence that interprofessional collaboration in primary care is frequently sustained by individual initiative rather than systemic design [42]. The *Perception* theme highlights that attitudes toward collaboration are strongly conditioned by personal exposure to the other profession. Physicians who had received chiropractic care or personally knew a chiropractor tended to describe more favourable views in their interviews, a pattern consistent with previous Canadian surveys [22] and with Myburgh and colleagues’ narrative review [8], which identified professional mistrust rooted in educational silos and historical tensions as a persistent barrier. Busse and colleagues [22] found that older physicians held more negative attitudes toward chiropractic, suggesting that newer cohorts may be less constrained by these historical tensions, a modest but important signal for the long-term trajectory of interprofessional relations. As Newell and colleagues [43] argue, targeted educational interventions can be a powerful catalyst for change. Kopansky-Giles and colleagues [44] showed that even brief, interprofessional training sessions significantly enhance collaborative competencies within primary-care teams.

The *Organization of care and services* theme reveals that structural conditions rarely match the collaborative intentions of individual practitioners. Out-of-pocket costs, exclusion from public care settings, and the absence of formalized referral mechanisms were identified as the primary structural impediments, a finding that resonates strongly with Mior et al.‘s earlier work [7] and with recent evidence from Australia [45]. While most participants endorsed the value of chiropractic integration into Family Medicine Groups, Busse and colleagues [22] remind us that physician support for formal integration and public funding remains far from unanimous. Previous reports suggested that integrating chiropractors in interprofessional teams is feasible and enhances collaboration [8, 10, 44, 45].

The *Communication* theme underscores that the quality of interprofessional exchanges is inseparable from the relational and structural conditions in which they occur. Face-to-face interaction was universally prized for its immediacy and capacity to resolve ambiguity, yet it depended almost entirely on physical proximity. A recurring finding was the asymmetry in communication initiation: chiropractors disproportionately bore the burden of initiating contact with physicians, a dynamic consistent with the broader literature documenting the marginal positioning of chiropractic within formal primary care networks [7, 8]. In our study, established professional relationships emerged as an important enabler of effective communication, a finding consistent with previous research in similar interprofessional contexts [8, 45]. Participants themselves identified investments in interoperable record systems, standardized referral templates, and protected time for case discussions as priority strategies to reduce this asymmetry and formalize what currently depends on individual goodwill.

### Implications for practice

Sustainable interprofessional musculoskeletal care in multidisciplinary settings requires action on three interconnected fronts. First, shared knowledge and mutual respect can be cultivated through embedding chiropractic content in medical curricula, joint continuing-education events, and collaborative research initiatives. Second, collaborative workflows might be formalized by integrating chiropractors into public clinic settings such as Family Medicine Groups, codifying clear referral pathways, and enabling chiropractors to complete the documentation required by workers’ compensation and automobile insurance boards for musculoskeletal conditions. Third, communication equity might be ensured through interoperable record systems, standardized referral templates, and protected time for interprofessional case discussions.

From a health-policy standpoint, our findings point to two levers that warrant formal evaluation. First, partial public funding or reimbursement parity for chiropractic musculoskeletal care would address the out-of-pocket costs and the reimbursement asymmetry relative to physiotherapy that participants identified as primary barriers. Second, formally integrating chiropractors into Family Medicine Groups, as participants favoured, would embed collaboration within existing primary-care structures rather than leaving it to individual initiative. Because these directions derive from a small, collaboration-oriented sample in a single province, they should be regarded as hypotheses for policy experimentation and evaluated through feasibility and outcome studies before broader implementation.

### Strengths and limitations

The present study has several strengths. The explanatory sequential mixed-methods design enabled integration across professions and methodological approaches, enhancing the credibility and depth of findings. Sampling both chiropractors and physicians reduced single-group bias, and participants were drawn from a variety of clinical settings. Findings are informative at the patient, professional, and health system levels, and are contextually relevant to Quebec’s primary care landscape.

However, important limitations must be acknowledged. Most critically, both samples were small and highly self-selected. All participants already worked in settings where both professions were present, and not a single physician endorsed negative views of chiropractic, a pattern that likely reflects a collaboration-oriented subset of professionals rather than broader professional sentiment. Two of the five physician participants were in a personal relationship with a chiropractor, which likely amplified the favourable attitudes observed. These characteristics constitute a best-case scenario for collaboration, and findings should not be interpreted as representative of physician-chiropractor relations in Quebec more generally. Further, Quebec’s distinct regulatory and funding frameworks may limit transferability to other Canadian provinces or healthcare systems. The qualitative component relied on a single focus group with chiropractors and five physician interviews; a single group cannot capture the full range of experiences, and thematic saturation was not reached, as new themes continued to emerge across interviews; the qualitative component is therefore exploratory rather than comprehensive. The differing recruitment modalities across the two surveys (email with reminders for chiropractors, a mailed questionnaire without reminders for physicians) may also have differentially affected participation. Regarding transferability, the perceptual and communicative barriers identified, including educational silos, technique heterogeneity, professional mistrust rooted in historical tensions, and reliance on informal, relationship-driven communication, resonate with findings from other Canadian provinces and from comparable health systems such as Australia [22], and from comparable health systems such as Australia [45], and may therefore be cautiously transferable to other developed countries with similar primary-care structures. In contrast, the structural and financial barriers, are shaped by Quebec’s specific regulatory and funding context. These are likely to be most transferable to jurisdictions where chiropractic remains outside publicly funded care, and least applicable where it is already integrated into and reimbursed by the public system. Confirmatory studies in other provinces and health systems would help delineate which determinants of physician-chiropractor collaboration are context-specific and which are more universal.

## Conclusion

In multidisciplinary clinic settings, interprofessional collaboration between physicians and chiropractors is broadly valued by both professional groups and perceived as beneficial for patient care, yet practice remains structurally underdeveloped. Collaboration depends almost entirely on individual initiative rather than formalized organizational frameworks. Improvements in education, organizational integration, and communication infrastructure are needed to translate this goodwill into consistent, high-quality interprofessional musculoskeletal care. Future research should build on these exploratory findings through feasibility studies testing specific collaborative models such as the integration of chiropractors into Family Medicine Groups, and mixed-methods longitudinal designs that capture how collaboration evolves over time and under varying structural conditions. Patient perspectives on referral decision-making and care experience within collaborative settings also remain an important gap to address.

## Supplementary Information

Below is the link to the electronic supplementary material.

Supplementary Material 2. Reporting of investigator reflexivity for the qualitative phase of our study.

Supplementary Material 1. Questionnaires and interview guides.

Supplementary Material 4. Manuscript revision checklist

Supplementary Material 3. Good Reporting of A Mixed Methods Study (GRAMMS) checklist.

## Data Availability

The data that support the findings of this study are not openly available but are available from the corresponding author upon reasonable request within 6 months of the manuscript publication. After that time period the data will be destroyed as specified within our ethical certification application.

## Declarations

### Use of artificial intelligence tools

During the preparation of this manuscript, the authors used Claude Sonnet 4.6 (Anthropic, 2026) to assist with text editing and language refinement. All content was subsequently reviewed and verified by the authors, who take full responsibility for the accuracy and integrity of the published work.
