# Supplementary material for: Characteristics of interprofessional collaboration between physicians and chiropractors within Québec multidisciplinary clinics: a mixed-methods study
Source: Chiropr Man Therap. 2026 Sep 5;34:48. doi: 10.1186/s12998-026-00673-9 (PMC13546507; doi:10.1186/s12998-026-00673-9)
Supplement: Supplementary file 1 [file 12998_2026_673_MOESM2_ESM.pdf]

Chiropractor Survey

SECTION 1 / 12 : SOCIODEMOGRAPHIC CHARACTERISTICS

At any time, you may stop completing the questionnaire and return to it later, on the same or a different computer. On each page of the questionnaire, your answers will be saved when you click the "next" button at the bottom of the page.

1.1. What is your gender?

- ■ Female
- ■ Male
- ■ Other

SECTION 2 / 12 : PROFESSIONAL PROFILE

2.1. How many years have you been practising chiropractic in Quebec?

[_____________]

2.2. In the context of your practice, do you have scheduled meetings to discuss clinical cases with other practitioners/professionals (e.g., interdisciplinary or multidisciplinary meetings)?

- ■ No
- ■ Yes
- ■ Yes, in the past

2.3. Have you ever taken a course or training specifically on interactions with other practitioners (e.g., on interdisciplinarity)?

- ■ No
- ■ Yes, during my basic chiropractic training
- ■ Yes, through continuing education
- ■ Other

2.4. What is your type of affiliation with the clinic?

- ■ Owner or co-owner
- ■ Self-employed worker renting space in the clinic
- ■ Employee or salaried worker

2.5. How many hours did you work in a typical work week over the past 12 months?

[_____________]

2.6. How many patients did you see in a typical work week over the past 12 months?

[_____________]

2.7. During the past 12 months, did you act as a clinical supervisor for chiropractic students (internship, practicum)?

- ■ No
- ■ Yes

2.8. Among the patients who consulted you during the past 12 months, approximately what percentage (%) was referred by a physician?

[_____________]

2.9. For the following questions, please indicate what percentage of your patients, during the past 12 months, had chiropractic treatments covered by...

2.10. The CNESST (workers' compensation board)?

- ■ 0–10%
- ■ 11–25%

- 26–50%
- 51–75%
- 76–100%

2.11. The SAAQ (automobile insurance board)?

- 0–10%
- 11–25%
- 26–50%
- 51–75%
- 76–100%

2.12. Another organization (e.g., Veterans Affairs, IVAC, etc.)?

- 0–10%
- 11–25%
- 26–50%
- 51–75%
- 76–100%

2.13. For the following questions, please indicate what percentage of your patients, during the past 12 months, consulted for a...

2.14. Acute condition (duration less than 4 weeks)?

- 0–10%
- 11–25%
- 26–50%
- 51–75%
- 76–100%

2.15. Sub-acute condition (duration 4 to 12 weeks)?

- 0–10%
- 11–25%
- 26–50%
- 51–75%
- 76–100%

2.16. Chronic condition (duration more than 12 weeks)?

- 0–10%
- 11–25%
- 26–50%
- 51–75%
- 76–100%

SECTION 3 / 12 : GENERAL OVERVIEW OF YOUR WORKPLACE

3.1. Where is your practice located?

- In your home
- In a building containing only the clinic/practice
- In a building containing multiple businesses or organizations, one of which has a health mandate
- In a building containing multiple businesses or organizations without a health mandate
- In a public health network facility (e.g., medical clinic/FMG, hospital)
- In an educational institution (e.g., CEGEP)
- Other

3.2. What is your remuneration model as a chiropractor at the clinic?

- Per patient (fee-for-service)

■ Hourly salary
■ Mixed model: salary and fee-for-service
■ Other

SECTION 4 / 12 : INTERACTIONS WITH OTHER PRACTITIONERS DURING THE PAST 12 MONTHS

4.1. In this section, we invite you to reflect on: your written, oral, virtual, or in-person interactions with other practitioners/professionals, including other chiropractors, who work in your clinic or elsewhere, and during the past 12 months.

4.2. During the past 12 months, how often did you use the following means to interact with other practitioners/professionals?

	Every day	Every week	Every month	A few times	Never
**4.3.** Phone discussion	■	■	■	■	■
**4.4.** Email	■	■	■	■	■
**4.5.** Letter/form transmitted via the patient	■	■	■	■	■
**4.6.** Letter/form sent by mail or fax	■	■	■	■	■
**4.7.** Message transmitted verbally via the patient	■	■	■	■	■
**4.8.** Unplanned in-person discussion (e.g., in the hallway)	■	■	■	■	■
**4.9.** Planned meeting in person with one or more practitioners to discuss clinical cases	■	■	■	■	■
**4.10.** Evaluation or intervention where you are present with a patient at the same time as one or more other practitioners (e.g., joint evaluation)	■	■	■	■	■

4.11. Other means used to interact and frequency of use:

[Open text field – max. 4,000 characters]

4.12. During the past 12 months, how often did you have written, oral, virtual, or in-person interactions with each of the following practitioners working in your clinic or elsewhere?

	Every day	Every week	Every month	A few times	Never
**4.13.** Family physician	■	■	■	■	■
**4.14.** Sports medicine physician	■	■	■	■	■
**4.15.** Neurologist	■	■	■	■	■
**4.16.** Orthopaedic surgeon	■	■	■	■	■
**4.17.** Rheumatologist	■	■	■	■	■

4.18. Other medical specialty(ies) and frequency of interactions:

[Open text field – max. 4,000 characters]

4.19. Please choose the number between 0 and 10 that best corresponds to your perceptions for each of the questions below, where 0 indicates not at all and 10 indicates completely. Thus, during the past 12 months...

	0	1	2	3	4	5	6	7	8	9	10
**4.20.** To what extent did you make efforts to avoid creating conflicts regarding the sharing of tasks and responsibilities with other practitioners?	■	■	■	■	■	■	■	■	■	■	■
**4.21.** To what extent were your professional relationships with the various practitioners equal?	■	■	■	■	■	■	■	■	■	■	■
**4.22.** To what extent was the sharing of responsibilities between you and other practitioners pre-established?	■	■	■	■	■	■	■	■	■	■	■
**4.23.** To what extent were your clinical activities with patients coordinated with the clinical activities of other practitioners with the same patients?	■	■	■	■	■	■	■	■	■	■	■
**4.24.** To what extent did you take into consideration the comprehensive needs of your patients, i.e., their physical, psychological, and social needs?	■	■	■	■	■	■	■	■	■	■	■
**4.25.** To what extent did you take into consideration data collected by other practitioners?	■	■	■	■	■	■	■	■	■	■	■
**4.26.** To what extent did you tolerate the existence of grey areas in the sharing of responsibilities with other practitioners?	■	■	■	■	■	■	■	■	■	■	■
**4.27.** To what extent did you develop common intervention plans with other practitioners?	■	■	■	■	■	■	■	■	■	■	■
**4.28.** To what extent did you seek advice or opinions from other practitioners?	■	■	■	■	■	■	■	■	■	■	■
**4.29.** To what extent did you exchange information about shared patients with other practitioners?	■	■	■	■	■	■	■	■	■	■	■
**4.30.** To what extent did you cooperate with other practitioners to ensure the follow-up of shared patients?	■	■	■	■	■	■	■	■	■	■	■
**4.31.** To what extent did you refer your patients to other practitioners?	■	■	■	■	■	■	■	■	■	■	■

4.32. During the past 12 months, on average how many minutes per week did you devote to your written, virtual, oral, or in-person interactions with other practitioners?

[______________]

4.33. During the past 12 months, for what percentage (%) of your patients did you have one or more interactions with other practitioners, whether written, oral, virtual, or in-person?

[______________]

4.34. Thinking about the interactions you had during the past 12 months with other practitioners, who most often initiated the interactions — you or the other practitioners?

■ Me
■ The other practitioners
■ Both me and the other practitioners equally
■ Not applicable

4.35. What is your level of satisfaction with the interactions you had during the past 12 months with:

	Completely satisfied	Rather satisfied	Neither satisfied nor dissatisfied	Rather dissatisfied	Completely dissatisfied	N/A

**4.36.** Family physicians	■	■	■	■	■	■
**4.37.** Specialist physicians	■	■	■	■	■	■
**4.38.** Other chiropractors	■	■	■	■	■	■
**4.39.** Other practitioners (e.g., physiotherapist, occupational therapist, kinesiologist)	■	■	■	■	■	■

SECTION 5 / 12 : PERCEPTIONS OF INTERACTIONS WITH OTHER PRACTITIONERS

For each of the following statements, please indicate your level of agreement.

	Strongly agree	Rather agree	Neither	Rather disagree	Strongly disagree
**5.1.** I have sufficient knowledge of the scopes of practice of other practitioners.	■	■	■	■	■
**5.2.** Other practitioners perceive chiropractors as professionals.	■	■	■	■	■
**5.3.** Other practitioners know what chiropractic is.	■	■	■	■	■

SECTION 6 / 12 : PERCEIVED EFFECTS OF YOUR INTERACTIONS

During the past 12 months, the interactions I had with other practitioners have:

	Strongly agree	Rather agree	Neither	Rather disagree	Strongly disagree
**6.1.** Improved the clinical condition of my patients	■	■	■	■	■
**6.2.** Increased patient satisfaction	■	■	■	■	■
**6.3.** Helped to better meet the biopsychosocial needs of my patients	■	■	■	■	■
**6.4.** Harmed the quality of services I provided to my patients	■	■	■	■	■
**6.5.** Increased my job satisfaction	■	■	■	■	■
**6.6.** Increased the number of chiropractic referrals at the clinic	■	■	■	■	■
**6.7.** Helped raise awareness of chiropractic among other practitioners	■	■	■	■	■

6.8. Other perceived effect(s):

[Open text field – max. 4,000 characters]

SECTION 7 / 12 : FACILITATORS AND BARRIERS TO INTERACTIONS WITH OTHER PRACTITIONERS

During the past 12 months, please specify to what extent the following elements represented a facilitator or a barrier to your interactions with other practitioners.

	Major fac ilitator	Minor fac ilitator	Neither	Minor barrier	Major barrier	N/A

**7.1.** Patient condition complexity	■	■	■	■	■	■
**7.2.** Personally knowing other practitioners	■	■	■	■	■	■
**7.3.** Your workload	■	■	■	■	■	■
**7.4.** The workload of other practitioners	■	■	■	■	■	■
**7.5.** Shortage of practitioners in your region	■	■	■	■	■	■
**7.6.** Your remuneration model	■	■	■	■	■	■
**7.7.** The precarious financial situation of certain patients	■	■	■	■	■	■
**7.8.** The proximity of other practitioners	■	■	■	■	■	■

7.9. Other element(s) — please specify if facilitator(s) or barrier(s):

[Open text field – max. 4,000 characters]

SECTION 8 / 12 : SOCIAL ACTIVITIES WITH OTHER PRACTITIONERS

The next section relates to your social activities with other practitioners. During the past 12 months, how often did you participate in social activities (e.g., lunch at or away from work, Christmas party, after-work gathering) with other practitioners...

	Every day	Every week	Every month	A few times	Never	N/A
**8.1.** From your clinic?	■	■	■	■	■	■
**8.2.** From outside your clinic?	■	■	■	■	■	■

SECTION 9 / 12 : RESOURCES AND OPERATING PROCEDURES AT THE CLINIC

9.1. At your clinic, do you have access to the following resources as a chiropractor?

	No	Yes
**9.2.** Computerized patient records for chiropractic	■	■
**9.3.** Professional email	■	■
**9.4.** Fax machine	■	■

9.5. For each of the following types of practitioners, please indicate the number who work in your clinic, including yourself (e.g., 0 or 3). Note: if a practitioner holds 2 positions in the clinic (e.g., physiotherapy technician and massage therapist), please count them in both places.

9.6. Family physician [_________]

9.7. Sports medicine physician [_________]

9.8. Neurologist [_________]

9.9. Orthopaedic surgeon [_________]

9.10. Rheumatologist [_________]

9.11. Other medical specialty — please specify type(s) and number of practitioners:

[Open text field]

9.12. For record-keeping, do you use shared patient records with one or more other types of practitioners at your clinic?

■ No

- ■ Yes

9.13. At your clinic, do you have forms for use in interactions with other practitioners (e.g., letter templates)?

- ■ No
- ■ Yes

9.14. At your clinic, do you have written or implicit operating rules regarding interactions with other practitioners or other health facilities (e.g., when to send a letter to the physician, service agreements for referrals to other clinics)?

- ■ No
- ■ Yes, written rules
- ■ Yes, implicit rules
- ■ Yes, both written and implicit rules
- ■ Other(s)

SECTION 10 / 12 : VISIONS AND VALUES PROMOTED IN YOUR CLINIC

On a scale of 0 to 10, where 0 indicates no importance and 10 indicates the greatest possible importance, what is the importance currently given in your clinic to the following elements:

	0	1	2	3	4	5	6	7	8	9	10
**10.1.** Physical health of patients	■	■	■	■	■	■	■	■	■	■	■
**10.2.** Psychological and social health of patients	■	■	■	■	■	■	■	■	■	■	■
**10.3.** Prevention and health promotion	■	■	■	■	■	■	■	■	■	■	■
**10.4.** Evidence-based practice (research findings)	■	■	■	■	■	■	■	■	■	■	■
**10.5.** Financial profitability	■	■	■	■	■	■	■	■	■	■	■
**10.6.** Respect, courtesy, and confidentiality	■	■	■	■	■	■	■	■	■	■	■
**10.7.** Interactions between chiropractors and other practitioners (from the clinic or outside)	■	■	■	■	■	■	■	■	■	■	■

SECTION 11 / 12 : PRACTICES AND SERVICES OFFERED AT THE CLINIC

11.1. Are the following chiropractic services offered at your clinic?

	No	Yes
**11.2.** Paediatrics	■	■
**11.3.** Perinatal care/obstetrics	■	■
**11.4.** Sports injury	■	■
**11.5.** Geriatrics	■	■
**11.6.** Occupational (CNESST, etc.)	■	■
**11.7.** Neurological conditions (vertigo, coordination disorders, etc.)	■	■
**11.8.** Generic spinal conditions (low back pain, neck pain, thoracic pain, etc.)	■	■
**11.9.** Headache/migraine	■	■
**11.10.** Peripheral conditions	■	■

**11.11.** Prevention/wellness	■	■

11.12. Other service(s):

[Open text field – max. 4,000 characters]

11.13. At your clinic, what is the average duration in minutes of initial chiropractic evaluations?

- ■ ≤5 minutes
- ■ 6–10 minutes
- ■ 11–15 minutes
- ■ 16–30 minutes
- ■ 31–45 minutes
- ■ 46–60 minutes
- ■ >60 minutes

11.14. At your clinic, what is the average duration in minutes of treatment sessions?

- ■ ≤5 minutes
- ■ 6–10 minutes
- ■ 11–15 minutes
- ■ 16–30 minutes
- ■ 31–45 minutes
- ■ 46–60 minutes
- ■ >60 minutes

SECTION 12 / 12 : COMMENTS

12.1. * Thank you for your valuable responses! Would you like to share any additional reflections or comments related to collaboration between chiropractors and physicians?

[Open text field – max. 4,000 characters]

12.2. Completion of this questionnaire provides a compensation of $50. To what address would you like us to send the compensation payment?

[Open text field – max. 4,000 characters]

12.3. * As part of the next step in this project, we would like to survey physicians who regularly collaborate with chiropractors. Could you provide us with the names (and contact details) of the physicians with whom you collaborate so that we may send them a questionnaire on interprofessional collaboration?

[Open text field – max. 4,000 characters]

12.4. * To further our understanding of physician-chiropractor collaboration, we would like to organize a focus group of chiropractors. Would you agree to be recontacted for this purpose?

- ■ No
- ■ Yes

Physician Survey

SECTION 1 / 5 : SOCIODEMOGRAPHIC CHARACTERISTICS

1.1. What is your gender?

- ■ Female
- ■ Male
- ■ Other

SECTION 2 / 5 : PROFESSIONAL PROFILE

2.1. How would you describe your professional activities (you may check more than one)?

- ■ Community
- ■ Hospital
- ■ Multidisciplinary
- ■ Private practice
- ■ Academic/teaching
- ■ Research
- ■ Other(s)

2.2. Is chiropractic care available at one of your practice locations?

- ■ Yes, at the same practice location
- ■ Yes, near the practice location
- ■ No
- ■ No, but I would be open to working with a chiropractor
- ■ Uncertain
- ■ I do not work in a multidisciplinary setting

2.3. Which best describes the proportion of your patients with musculoskeletal chief complaints?

- ■ 0% to 25%
- ■ 25% to 50%
- ■ 50% to 75%
- ■ 75% to 100%

2.4. Which best describes your area of clinical interest (please check all that apply)?

- ■ General family medicine
- ■ Emergency medicine
- ■ Anaesthesia
- ■ Psychotherapy/psychiatry
- ■ Pain medicine
- ■ Obstetrics and gynaecology
- ■ Occupational medicine
- ■ Geriatrics
- ■ Sports medicine
- ■ Paediatrics
- ■ Palliative care
- ■ Orthopaedics
- ■ Musculoskeletal medicine/physiatry
- ■ Rheumatology
- ■ Neurology
- ■ Other

2.5. How many years have you been in practice?

- ■ Less than 5 years
- ■ 5 to 10 years
- ■ 11 to 20 years
- ■ More than 20 years

SECTION 3 / 5 : QUESTIONS ON CHIROPRACTIC

3.1. How would you rate your knowledge of chiropractic?

- ■ No knowledge
- ■ Little knowledge
- ■ Moderate knowledge
- ■ Very extensive knowledge

3.2. Have you ever received chiropractic care?

- ■ Yes
- ■ No

3.3. During your initial medical training, were you presented with information on chiropractic?

- ■ Yes, and the information was generally favourable
- ■ Yes, and the information was generally neutral
- ■ Yes, and the information was generally unfavourable
- ■ No

3.4. In your opinion, should information on chiropractic be presented during initial medical training?

- ■ Yes, definitely
- ■ Possibly
- ■ Uncertain
- ■ Probably not
- ■ No, definitely not

3.5. Are you comfortable discussing chiropractic care with your patients?

- ■ Yes, definitely
- ■ Quite
- ■ Uncertain
- ■ Probably not
- ■ No, definitely not

3.6. Are you interested in learning more about chiropractic?

- ■ Yes
- ■ No
- ■ Uncertain

3.7. How did your opinion on chiropractic develop? (please check all that apply)

- ■ Personal treatment experience
- ■ Family and friends
- ■ Research literature
- ■ Media
- ■ Medical school
- ■ Patient feedback
- ■ Professors/supervisors/mentors
- ■ Relationship with a chiropractor
- ■ Internship
- ■ I have no opinion on chiropractic

- ■ Other

3.8. At what point did your opinion on chiropractic primarily develop?

- ■ Before medical school (faculty of medicine)
- ■ During medical school
- ■ After medical school

3.9. Should chiropractic care be available in a multidisciplinary environment?

- ■ Yes
- ■ No
- ■ Uncertain

3.10. Should chiropractic care be covered, in whole or in part, by public/government funding?

- ■ Yes
- ■ No
- ■ Uncertain

3.11. How often do you refer patients to chiropractic care?

- ■ Every day
- ■ Every week
- ■ Every month
- ■ Every year
- ■ Never

3.12. How many patients do you refer to chiropractic care in a typical year?

- ■ None
- ■ 1 to 10
- ■ 11 to 25
- ■ 26 to 50
- ■ More than 50

3.13. If you refer patients to chiropractic care, what motivates your referrals? (please check all that apply)

- ■ Patient request
- ■ Non-response to medical treatment
- ■ The literature supports chiropractic care for certain conditions
- ■ Relationship with a particular chiropractor
- ■ My own positive experience as a patient receiving chiropractic care
- ■ I do not refer patients to chiropractic care
- ■ Other

3.14. For which condition(s) do you commonly refer patients to chiropractic care?

[Open text field – max. 4,000 characters]

3.15. Does the diversity of approaches within the chiropractic profession present a barrier to greater collaboration with physicians?

- ■ Yes
- ■ No
- ■ Uncertain

3.16. Do you perceive chiropractors as primary-care professionals?

- ■ Yes, definitely
- ■ Rather
- ■ Uncertain
- ■ Probably not
- ■ No, definitely not

3.17. Would you like to receive a consultation note from a chiropractor who has seen one of your patients?

- ■ Yes
- ■ No
- ■ Uncertain
- ■ Not applicable in the context of my practice

3.18. Have patients been referred to you by chiropractors in order for you to refer them for imaging studies?

- ■ Yes
- ■ No
- ■ Not applicable in the context of my practice

3.19. In your opinion, adverse effects associated with chiropractic care are frequent:

- ■ No
- ■ Uncertain
- ■ Yes
- ■ Yes, but serious events are rare

3.20. In your opinion, what level of education is required to obtain a chiropractic licence?

- ■ College technical diploma
- ■ Bachelor's degree
- ■ First-cycle doctorate (DC)
- ■ Master's degree
- ■ PhD

3.21. For each of the following statements, indicate your level of agreement.

	Strongly agree	Agree	Undecided	Disagree	Strongly disagree
**3.22.** Chiropractors recommend unnecessary treatment plans	■	■	■	■	■
**3.23.** Chiropractic care is effective for certain neuromusculoskeletal conditions	■	■	■	■	■
**3.24.** Chiropractors make excessive use of imaging	■	■	■	■	■
**3.25.** Chiropractors provide a patient-centred approach	■	■	■	■	■
**3.26.** When I see patients who have seen a chiropractor, I often need to spend time correcting misinformation they have received	■	■	■	■	■
**3.27.** Spinal manipulation of the cervical region is a generally safe intervention for patients	■	■	■	■	■
**3.28.** Chiropractic care is effective for certain non-musculoskeletal conditions	■	■	■	■	■
**3.29.** The family physician assumes professional liability when referring a patient to a chiropractor	■	■	■	■	■
**3.30.** Chiropractors can reduce physician overload for musculoskeletal complaints	■	■	■	■	■
**3.31.** Chiropractors provide patients with misinformation about vaccination	■	■	■	■	■
**3.32.** Chiropractic care is an effective treatment for post-surgical rehabilitation	■	■	■	■	■

**3.33.** Chiropractors lack sufficient clinical training	■	■	■	■	■
**3.34.** Chiropractic care is a useful supplement to conventional medicine	■	■	■	■	■
**3.35.** Chiropractors engage in overly aggressive marketing practices	■	■	■	■	■
**3.36.** Chiropractic includes notions and practices from which medicine could benefit	■	■	■	■	■
**3.37.** The effects of chiropractic manipulation are due to a placebo effect	■	■	■	■	■
**3.38.** Chiropractors practise in accordance with evidence-based practice	■	■	■	■	■
**3.39.** Chiropractic has no place in the routine care of my patients	■	■	■	■	■
**3.40.** Chiropractic creates dependency on care in patients who receive short-term symptom relief	■	■	■	■	■

3.41. Overall, my impression of chiropractic is:

	Very good	Good	Undecided	Poor	Very poor
**3.41.** Overall impression of chiropractic	■	■	■	■	■

SECTION 4 / 5 : PERCEPTIONS OF COLLABORATION

4.1–4.5. For each of the following statements, indicate your level of agreement.

	Strongly agree	Rather agree	Neither	Rather disagree	Strongly disagree
**4.1.** You are satisfied with the collaboration with chiropractors in your practice setting.	■	■	■	■	■
**4.2.** Collaboration with chiropractors requires a significant investment of time.	■	■	■	■	■
**4.3.** Chiropractors provide relevant suggestions for patient management.	■	■	■	■	■
**4.4.** Your collaboration with chiropractors has allowed you to develop your knowledge of chiropractic care.	■	■	■	■	■
**4.5.** In the future, you wish to continue collaborating with chiropractors in the same or a similar way.	■	■	■	■	■

4.6. For each of the following statements, please indicate your level of agreement. *Collaboration and my interactions with chiropractors:*

	Strongly agree	Rather agree	Neither agree nor disagree	Rather disagree	Strongly disagree
**4.7.** Improves the health condition of my patients	■	■	■	■	■
**4.8.** Helps clarify the diagnosis of my patients' chief complaints	■	■	■	■	■
**4.9.** Improves the satisfaction of my patients	■	■	■	■	■

**4.10.** Helps me determine relevant diagnostic tests for my patients	■	■	■	■	■
**4.11.** Helps me determine the patient's care trajectory (other practitioner, specialist, or service)	■	■	■	■	■
**4.12.** Reduces my workload	■	■	■	■	■
**4.13.** Improves the quality of services my patients receive	■	■	■	■	■

4.14. Do you perceive any drawbacks to collaborating with chiropractors that we have not mentioned?

[Open text field – max. 4,000 characters]

4.15. Has collaboration with chiropractors brought benefits that we have not mentioned?

[Open text field – max. 4,000 characters]

4.16. In your opinion, what is the main barrier to integrating chiropractors into care teams?

[Open text field – max. 4,000 characters]

4.17. Where do you believe chiropractors should be integrated into the health network (multiple choice)?

- ■ Specialized clinic (tertiary care)
- ■ Hospital outpatient clinic
- ■ Emergency department
- ■ FMG/medical clinic
- ■ CLSC (community health centre)
- ■ CHSLD (long-term care facility)
- ■ Rehabilitation centre
- ■ Chiropractors have no place in the health network
- ■ Other, please specify:

SECTION 5 / 5 : COMMENTS

5.1. Do you have any other comments about collaboration between physicians and chiropractors?

[Open text field – max. 4,000 characters]

5.2. Completion of this questionnaire provides a compensation of $50. To what address would you like us to send the compensation payment?

[Open text field – max. 4,000 characters]

5.3. To further our understanding of physician-chiropractor collaboration, we would like to organize individual interviews (compensation of $100). Would you agree to be recontacted for this purpose?

- ■ Yes
- ■ No

Focus Group Guide – Chiropractors

INTERVIEWER INTRODUCTION

Hello, my name is _____________. I have been tasked with exploring interactions between physicians and chiropractors that could improve interprofessional collaboration and patient care.

During this discussion, I would like us to address the following themes: the advantages, disadvantages, barriers, facilitators, and impacts of collaboration with physicians.

Advantages and disadvantages of collaboration with physicians.

Main questions	Supplementary questions	Clarifying questions
■ Can you tell me about the advantages you have observed when collaborating with physicians?<br>■ What advantages would you like to have when collaborating?	■ From your point of view?<br>■ From your patients' point of view?<br>■ From the physicians' point of view?	■ Could you tell me a little more?<br>■ Could you elaborate?<br>■ Could you give some examples?
■ Based on your experience, what are the disadvantages related to collaboration between physicians and chiropractors?	■ From your point of view?<br>■ From your patients' point of view?<br>■ From the physicians' point of view?	
■ How would you like to collaborate?<br>■ What roles would you like to have?	■ For your patients?<br>■ For your practice?<br>■ For the health system?	

BARRIERS AND FACILITATORS

Main questions	Supplementary questions	Clarifying questions
■ In general, what are the barriers to collaboration between physicians and chiropractors?	■ From your point of view?<br>■ From your patients' point of view?<br>■ From the physicians' point of view?	■ Could you tell me a little more?<br>■ Could you elaborate?<br>■ Could you give some examples?
■ What factors would facilitate interprofessional collaboration?<br>■ What improvements would you like to see in the collaboration between physicians and chiropractors?		

IMPACTS

Main questions	Supplementary questions	Clarifying questions

■ In your opinion, what are the positive impacts of collaboration between physicians and chiropractors?<br>■ In your opinion, what are the negative impacts of collaboration between physicians and chiropractors?	■ For your patients?<br>■ For you?<br>■ For the physicians?<br>■ For the health system?	■ Could you tell me a little more?<br>■ Could you elaborate?<br>■ Could you give some examples?

CLOSING

■ *OR*	■ Do you have a

Individual Interview Guide – Physicians

INTRODUCTION

• Acknowledgements
• Explanation of the interview process:
  – Duration of approximately 60 minutes; approximately 6 to 10 individual interviews with physicians collaborating with chiropractors
  – Interviewee's role: share your views and knowledge — there are no right or wrong answers; each person's opinion is valuable
  – Interviewer's role: ask questions and request clarifications, rephrase questions as needed
  – Reminder of data confidentiality
  – Confirm consent to record
  – Any questions before we begin?

Theme 1: Professional Situation

Question 1: To begin, can you describe your current work?

- Full-time/part-time
- Patient population
- Type of practice
- Other functions beyond that of physician?
- Other workplaces?

Question 2: What was your career path before working here?

- University training
- Professional experience
- Continuing education

Question 3: Tell me about your work environment.

- Patient population
- Services offered
- Staff
- Resources
- Remuneration model
- Unidisciplinary, multidisciplinary teamwork
- Physical spaces (internal and external) — meeting space
- Other nearby health organizations (clinics, hospital, etc.)
- Values and objectives promoted
- Vision for care/services and for interactions
- Rules to follow (e.g., training policies, service policies — modes of intervention, treatment frequency)
- Service agreements (other facilities, CNESST/SAAQ)

Theme 2: Your Interactions with Chiropractors

I would now like you to tell me more specifically about your experiences of interactions with chiropractors.

Question 4: First, can you describe the interactions you have with chiropractors in the context of your work?

Question 5: In what contexts do these interactions take place?

- E.g., teamwork, memorandum of agreement
- Who initiates?
- Objectives
- Examples

Question 6: Describe the means by which you interact with chiropractors.

- E.g., prescription, referral (paper)
- Telephone
- Electronic
- Face-to-face
- Through an intermediary
- Fax
- Videoconference
- What are the advantages and disadvantages of each modality?

Question 7: Can you tell me who the chiropractors are with whom you interact in the context of your work?

Question 8: Tell me about your interactions with Mr./Ms./Dr. ______. How does it go?

- Frequency
- Timing
- Duration
- Interaction modes (phone, face-to-face, email, etc.)
- Initiators
- Formal/informal
- Teamwork/work team
- Reasons motivating the interactions
- Shared activities
- Content of exchanges (treatment plan, interventions, examinations, etc.)

Question 9: Can you describe examples of situations where you are not interacting with a chiropractor but you feel that it would be desirable?

Question 10: Can you describe cases or situations where you feel it is not necessary to interact with a chiropractor when you are treating patients with low back pain?

- E.g., acute, sub-acute, chronic
- With or without compensation/litigation
- With or without comorbidities
- Complexity
- Prognosis
- Direct access
- An example

Theme 3: Effects of Interactions with Other Professionals

I would now like you to talk about the effects of your interactions with chiropractors.

Question 11: In your opinion, what are the (positive and/or negative) effects of your interactions with chiropractors?

- For the patients who consult?
- More specifically, patients with low back pain?

- For chiropractors and other health professionals?
- For the clinic?
- For the health services offered?
- For the health system?
- Other effects?
- Examples / underlying mechanisms

Question 12: What are the (positive and/or negative) effects of not having interactions with chiropractors?

- How would your work proceed if you had no interactions with chiropractors?
- What would be the impacts on your work if you had no interaction?

Theme 4: Factors Influencing Your Interactions with Other Professionals

In the final section, I would like to discuss with you the factors that influence your interactions with chiropractors.

Question 13: How do you perceive the current situation in your work in terms of interactions with chiropractors?

- Is the degree of interaction adequate?

Question 14: In your opinion, what factors encourage or facilitate your interactions with chiropractors? In what way are these factors facilitating?

- E.g., in relation to patients with low back pain
- The chiropractors themselves
- Work settings and their internal rules (e.g., agreements, opening hours, remuneration model) — organizational incentives
- Systems, e.g., education system, professional system, health system
- Direct access
- The most important ones?
- Specific incentives?
- Reasons / underlying mechanisms

Question 15: What factors hinder or prevent interactions with chiropractors? In what way do these factors hinder?

Question 16: Can you name chiropractors with whom you do not interact? What are the reasons for that?

Question 17: How do you think things work in other private clinics?

Question 18: What could be done in the future to change/improve the current situation? What could your role be?

Conclusion

Question 19: To conclude, how do you think interactions between physicians and chiropractors should evolve in the future?

- In the best of all possible worlds, what should interactions between physicians and chiropractors look like?

You have raised truly interesting points and I thank you for that. Are there any other elements that have not been addressed and that you would like to bring up? Any final thoughts?

Do you have any questions?

Would you agree to be recontacted if necessary to validate information collected in the context of today's interview?   Yes _____   No _____

On behalf of all the members of the research team, I sincerely thank you for helping us in the completion of this research project. Your collaboration is very valuable to us!

Post-Interview Reflection Notes

Duration:

Description of the interview setting:

Persons present (without names):

Summary of content:

Interview dynamics (role of interviewer, outward signs from the interviewee — tone, gestures, movements, etc.):

Reflections:

Other notes:
