# Supplementary material for: Characteristics of interprofessional collaboration between physicians and chiropractors within Québec multidisciplinary clinics: a mixed-methods study
Source: Chiropr Man Therap. 2026 Sep 5;34:48. doi: 10.1186/s12998-026-00673-9 (PMC13546507; doi:10.1186/s12998-026-00673-9)
Supplement: Supplementary file 2 [file 12998_2026_673_MOESM1_ESM.docx]

| **Research Team and Reflexivity ^a^** | **Description** |
| --- | --- |
| *Personal Characteristics* | |
| Interviewer | Focus group with chiropractors was facilitated by MAB. Individual semi-structured interviews with physicians were conducted by MAB. |
| Credentials | DC, PhD |
| Occupation | Researcher/professor within the chiropractic department of UQTR |
| Gender | Male |
| Experience and training | MAB completed a PhD in Public Health with a specialty in epidemiology. While primarily trained in quantitative epidemiological methods, this study represented one of his first projects conducting qualitative interviews and focus groups. Prior to data collection, MAB reviewed qualitative research methodology literature and consulted with experienced qualitative researchers to ensure appropriate interview techniques. |
| *Relationship with Participants* | |
| Relationship established | Participants were recruited through professional networks and responded to study invitations. No prior relationship was established with participants before the study commenced with the exception of one participant from the focus group with whom the interviewer completed his chiropractic training. |
| Participant knowledge of the interviewer | Participants knew the interviewer was a researcher from the UQTR chiropractic department conducting a study on interprofessional collaboration between physicians and chiropractors. |
| Interviewer characteristics | The interviewer was a chiropractor and researcher, which may have influenced participants' responses, among chiropractor participants who might have felt more comfortable sharing experiences with someone from their profession, and among physician who might have felt more inclined to present the positive aspects of their collaboration. This potential bias was considered during data analysis. |

^a^ Adapted from the Consolidated Criteria for Reporting Qualitative Research (COREQ): <https://academic.oup.com/intqhc/article/19/6/349/1791966>
