# Supplementary material for: Characteristics of interprofessional collaboration between physicians and chiropractors within Québec multidisciplinary clinics: a mixed-methods study
Source: Chiropr Man Therap. 2026 Sep 5;34:48. doi: 10.1186/s12998-026-00673-9 (PMC13546507; doi:10.1186/s12998-026-00673-9)
Supplement: Supplementary file 4 [file 12998_2026_673_MOESM4_ESM.docx]

**Supplementary File 3. Good Reporting of A Mixed Methods Study (GRAMMS) checklist.**

*Criteria from O’Cathain A, Murphy E, Nicholl J. The quality of mixed methods studies in health services research. J Health Serv Res Policy. 2008;13(2):92–98. Completed for the present study.*

| **No.** | **GRAMMS criterion** | **How and where reported in this study** |
| --- | --- | --- |
| 1 | Describe the justification for using a mixed methods approach to the research question | Reported. The Introduction and Study Design explain that a mixed methods approach was used to combine quantitative survey data on the frequency, modalities, and perceived value of physician–chiropractor collaboration with qualitative data explaining the mechanisms, meanings, and contextual determinants underlying these patterns. This combination was needed to identify facilitators and barriers that neither strand could capture alone. (Introduction; Methods, Study Design.) |
| 2 | Describe the design in terms of the purpose, priority and sequence of methods | Reported. An explanatory sequential mixed methods design was used. The quantitative surveys were conducted and analysed first; the qualitative strand (one chiropractor focus group and five physician interviews) was then used to explain and deepen the survey findings, with the physician interview guide refined in light of the survey results. The qualitative strand thus served an explanatory role relative to the quantitative strand, which had temporal priority. (Methods, Study Design.) |
| 3 | Describe each method in terms of sampling, data collection and analysis | Reported. (Methods: Study Population and Recruitment; Data Collection; Analysis.)  **Quantitative.** Online surveys of chiropractors (n = 23) and physicians (n = 19) practising in multidisciplinary clinics; analysed with descriptive statistics; reported in line with STROBE.  **Qualitative.** One virtual semi-structured focus group with chiropractors (n = 7) and individual semi-structured interviews with physicians (n = 5), conducted via Microsoft Teams with a guide adapted from Perreault (2013); transcripts analysed by inductive (de novo) thematic analysis by two coders following Braun and Clarke; reported in line with COREQ. |
| 4 | Describe where integration has occurred, how it has occurred and who has participated in it | **Where.** At the interpretation and reporting stages (Methods, Mixed-Methods Integration and Reporting; Results, Integration of Quantitative and Qualitative Findings).  **How.** Through a joint display (Table 8) organized by meta-inference: for each integrated dimension the quantitative result and the corresponding qualitative finding were placed side by side, and a meta-inference was generated characterizing the fit between strands as confirmation, expansion, or discordance (Fetters, Curry & Creswell 2013; Guetterman, Fetters & Creswell 2015; O’Cathain, Murphy & Nicholl 2010).  **Who.** The meta-inferences were generated by the first author (MAB); the integrated interpretation was reviewed by the co-authors. |
| 5 | Describe any limitation of one method associated with the presence of the other method | Reported. The Strengths and Limitations section notes that both the survey and qualitative samples were small, self-selected, and drawn from the same collaboration-oriented multidisciplinary settings, so this selection limitation is common to both strands. Because the design was sequential, the physician interview guide was adapted from the survey findings, which may have focused the qualitative exploration on survey-derived issues and constrained the emergence of unanticipated themes. The differing recruitment modalities of the two surveys may also have affected participation. (Strengths and Limitations.) |
| 6 | Describe any insights gained from mixing or integrating methods | Reported. The Results subsection Integration of Quantitative and Qualitative Findings, the Discussion, and Table 8 present insights available only from integration: the qualitative data reframed physicians’ split ratings on cervical-manipulation safety (37% agreeing it is safe) as a remediable information deficit rather than a rejection of evidence; explained the coexistence of high perceived value (84% reporting improved care) with minimal formal structure as reliance on informal, relationship-driven collaboration; converted an abstract financial barrier into a concrete equity concern; and revealed a discordance between physicians’ self-rated patient-centredness (90%) and a coordination burden frequently shifted onto patients. |
